# A ring trial to harmonize *Toxoplasma gondii* microsatellite typing: comparative analysis of results and recommendations for optimization

**DOI:** 10.1007/s10096-023-04597-7

**Published:** 2023-04-24

**Authors:** M. Joeres, G. Cardron, K. Passebosc-Faure, N. Plault, M. Fernández-Escobar, C. M. Hamilton, L. O’Brien-Anderson, R. Calero-Bernal, L. Galal, C. Luttermann, P. Maksimov, F. J. Conraths, M. L. Dardé, L. M. Ortega-Mora, P. Jokelainen, A. Mercier, G. Schares

**Affiliations:** 1 Institute of Epidemiology, Federal Research Institute for Animal Health, Friedrich-Loeffler-Institut, Südufer 10, 17493 Greifswald-Insel Riems, Germany; 2 Centre National de Référence (CNR) Toxoplasmose Centre Hospitalier-Universitaire Dupuytren, Limoges, France; 3 Inserm U1094, IRD U270, Univ. Limoges, CHU Limoges, EpiMaCT - Epidemiology of Chronic Diseases in Tropical Zone, Institute of Epidemiology and Tropical Neurology, OmegaHealth, Limoges, France; 4 SALUVET, Animal Health Department, Faculty of Veterinary Sciences, Complutense University of Madrid, Madrid, Spain; 5 The Moredun Research Institute, Penicuik, Midlothian, UK; 6 Infectious Disease Preparedness, Statens Serum Institut, Copenhagen, Denmark; 7 Institute of Immunology, Federal Research Institute for Animal Health, Friedrich-Loeffler-Institut, -Insel Riems, Greifswald, Germany

**Keywords:** DNA quantification, Interlaboratory comparison, Toxoplasmosis, Genotyping, Subtyping

## Abstract

A ring trial among five European laboratories was organized to reach consistency in microsatellite (MS) typing of the zoonotic parasite *Toxoplasma gondii*. Three sample sets were circulated and analyzed by each laboratory following a previously published method that is based on fragment length polymorphism of 15 MS markers. The first sample set compared typing results in general and focused on effects of DNA concentration; the second sample set focused on the polymorphic fingerprinting markers that can differentiate *T. gondii* strains within the same archetypal lineage; and the third set focused on non-archetypal genotypes. Methodological variations between laboratories, including the software programs used to determine MS fragment length, were collated using a questionnaire. Overall, lineage-level typing results reached a high level of agreement, especially in samples with the highest DNA concentrations. However, laboratory-specific differences were observed for particular markers. Major median differences in fragment length, of up to 6 base pairs, were related to the fluorophore used to label fragment-specific primers. In addition, primer pairs with identical sequences obtained from different suppliers resulted in fragments of differing length. Furthermore, differences in the way the sequencing profiles were assessed and interpreted may have led to deviating results in fragment length determination. Harmonization of MS typing, for example, by using the same fluorophores or by numerical adjustments applied to the fragment-lengths determined, could improve the uniformity of the results across laboratories. This is the first interlaboratory comparison, providing guidelines (added as a supplement) for the optimization of this technique.

**Supplementary Information:**

The online version contains supplementary material available at 10.1007/s10096-023-04597-7.

## Introduction

*Toxoplasma gondii* is a zoonotic protozoan parasite that uses domestic cats and other felids as definitive hosts and causes clinical disease in both humans and animals [1–4]. It was recently ranked second out of 24 important foodborne parasites in Europe [5–7].

Globally, *T. gondii* has a complex population structure [8]. While populations of this parasite in many regions of the world belong to few clonal lineages [9], those observed in South America are much more diverse [8, 10].

A frequently used genotyping technique targets microsatellite (MS) sequences [11]. MS sequences are ubiquitous and polymorphic in the genomes of virtually all organisms [12]. For *T. gondii* typing, usually a set of up to 15 markers located on 11 different chromosomes of the *T*. *gondii* genome is used, including eight lineage typing markers (B18, M33, TUB2, XI.1, TgM-A, W35, IV.1, and B17) and seven fingerprinting markers (N61, M48, N83, N82, N60, M102, and AA). Fingerprinting markers are more polymorphic and were shown to resolve different isolates, applicable to both archetypal (type I, II, or III) and non-archetypal lineages [11].

MS sequences need to be amplified by multiplex or singleplex PCR using primer pairs, with one of the primers per pair labeled by a fluorophore. Subsequently, amplicons are separated on a capillary sequencer, including a size standard in each run, which allows determination of the lengths of the amplified MS fragments. Usually, three different fluorophores are used, which allows examination of a larger number of amplified fragments simultaneously in a single run on the capillary sequencer [11].

Another frequently used technique to type *T. gondii* is PCR-restriction fragment length polymorphism (PCR-RFLP), which can resolve *T. gondii* genotypes, but is—in contrast to MS typing—less suitable for differentiating parasites of the same lineage. PCR-RFLP *T. gondii* typing involves multiplex or singleplex PCR to amplify up to 11 markers, which are distributed over eight chromosomes, and the apicoplast [13].

Multilocus MS typing is used by many laboratories around the world [9, 14–17]. It is largely unknown, however, to which extent the lineage typing and fingerprinting results obtained by different laboratories are comparable. This is a challenge as a One Health approach, e.g., combining larger data sets on *T. gondii* genotypes from different sectors and across countries, which is needed to better understand the molecular epidemiology and transmission pathways of *T. gondii.*

To evaluate consistency in *T. gondii* MS typing, a ring trial was established among five European laboratories. Laboratories had different levels of experience with this typing technique, had slightly modified the original protocol, and used—at least in part—different laboratory equipment, reagents, and software. This ring trial led to the identification of major reasons for differences in MS typing. The results were used to establish harmonized guidelines for laboratories on implementing MS typing of *T. gondii*.

## Materials and methods

### Participating laboratories

Five European laboratories (A–E) participated. One laboratory (B) had previously established and published a MS typing method and served as the reference laboratory [11]. Another laboratory (C) had introduced the technique 4 years ago, one laboratory (E) 2 years ago, and the two remaining laboratories (A and D) very recently. Laboratory E organized sets of samples, shipment, and collection of results.

### Origin of samples

The ring trial was divided into three consecutive parts. The first part was planned to assess the capacity to type archetypal lineages of *T. gondii*, types I, II, and III, and evaluate the effect of DNA concentration on the accuracy of results. The samples comprised DNA aliquots collected from reference strains belonging to the three lineages types I, II, and III with RH [18], ME49 [19], and NED [20], respectively, in different dilutions. In addition, a sample from a type II × III recombinant strain (D200273; DNA of the *T. gondii* isolate TGA32090; provided by the Biological Resource Centre (BRC) Toxoplasma [http://www.toxocrb.com/]) was included (Table 1). Samples with the two highest DNA concentrations were provided only once, while the three lowest concentrations were provided two to four times in the panel.

**Table 1:** Composition of the sample set of the first part of the ring trial on microsatellite typing of *Toxoplasma gondii*

**Concentration**	**Sample dilution in bovine carrier DNA**				**Number of replicas**
	**RH (10 ng/μL)**	**ME49 (10 ng/μL)**	**NED (10 ng/μL)**	**D200273**^**a**^ **(10 ng/μL)**	
Dilution 1	10^−1^	10^−1^	10^−1^	10^−1^	1
Dilution 2	10^−2^	10^−2^	10^−2^	10^−2^	1
Dilution 3	10^−3^	10^−3^	10^−3^	10^−3^	2
Dilution 4	10^−4^	10^−4^	10^−4^	10^−4^	4
Dilution 5	10^−5^	10^−5^	10^−5^	10^−5^	4
Negative control	Bovine carrier DNA (100 ng/μL)				2

^a^D200273: DNA of the *T. gondii* isolate TGA32090; provided by the Biological Resource Centre (BRC) Toxoplasma

The second part was planned to assess the ability to discriminate different *T. gondii* type II strains using fingerprinting markers (Table 2). The DNA samples corresponded to 10 different *T. gondii* type II isolates, as confirmed by PCR-RFLP, and were provided in duplicate in the panel. Concentrations of DNA were adjusted so that they were similar to the 10^−1^ dilution of the first part.

**Table 2:** Composition of the sample sets of the second and third parts of the ring trial on microsatellite typing of *Toxoplasma gondii*

**Sample no. in second part and third part of the ring trial**	**DNA identifier of providing laboratory**	**Ct value at laboratory E**	**BRC** ^**a**^ **identifier**	**Sample ID, alternative name**	**Country of origin**	**Host**	**References**	**Type, ToxoDB# as determined by PCR-RFLP in laboratory E**	**Number of replicas**
2-1	D200109	18.22	NA	V15-2, E-EU-ELP-5	Czech Republic	Tiger	This study	Type II, #3	2
2-2	D200111	18.29	NA	V17-1, GER-EJP-10	Germany	Wild boar	[21]	Type II, #3	2
2-3	D200113	18.43	NA	P17/2479, GER-EJP-7	Germany	Cat	This study	Type II, #3	2
2-4	D200114	17.28	NA	P17/2480, GER-EJP-6	Germany	Cat	This study	Type II, #3	2
2-5	D200118	19.50	NA	V16-4, W-EU-EJP-1	Austria	Cat	This study	Type II, #1	2
2-6	D200119	20.19	NA	V16-5, GER-EJP-8	Germany	Cat	This study	Type II, #3	2
2-7	D200121	19.02	NA	V16-7, W-EU-EJP-2	Austria	Cat	This study	Type II, #1	2
2-8	D200127	17.52	NA	V30-3, GER-EJP-3	Germany	Chicken	This study	Type II, #1	2
2-9	D200129	18.27	NA	V87-2, E-EU-EJP-3	Czech Republic	Wildcat	This study	Type II, #3	2
2-10	D212556	19.54	NA	V10-1, GER-EJP-1	Germany	Deer	[21]	Type II, #3	2
2-11	Negative control, Bovine carrier DNA	NA	Not applicable	Not applicable	Not applicable	Not applicable	Not applicable	Not applicable	1
3-1	D221394	20.27	TgA119002	BENIN02, P19S1AJ6	Benin	Chicken	[22]	Africa 1	2
3-2	D221396	19.94	TgA105034	GABON03; GAB1-FEL-CAT001	Gabon	Cat	[23]	III	2
3-3	D221397	19.19	TgA105033	GABON02, GAB1-2007-CAP-AEG004	Gabon	Goat	[23]	III variant	2
3-4	D221398	18.40	TgH19006A	FRENCHGUIANA15, GUYS006-BAY	French Guiana	Human	[24]	Amazonian	2
3-5	D221399	19.32	TgH18013A	FRENCHGUIANA11, GUY013-2004-LAB	French Guiana	Human	[24]	Amazonian	2
3-6	D221400	18.52	TgH40002A	GUADELOUPE02, PAP002-2010-GOM	Guadeloupe	Human	[23]	Caribbean 2	2
3-7	D221401	17.73	TgA18009	FRENCHGUIANA01, GUY-CAN-FAM-009 (CH29)	French Guiana	Dog	[23]	Caribbean 1	2
3-8	D221402	18.38	TgH16012A	MARTINIQUE03, FDF012-MAN	Martinique	Human	[23]	Caribbean 3	2
3-9	D221403	19.19	TgA117041	SENEGAL17, 160622Gdom02	Senegal	Chicken	[23]	II ^b^	2
3-10	Negative control, bovine carrier DNA	NA	Not applicable	Not applicable	Not applicable	Not applicable	Not applicable	Not applicable	1

^a^Biological Resource Centre (BRC) Toxoplasma. ^b^Designated as type II in literature although W35 showed a variation from type II pattern (244 bp instead of 242)

Finally, the last part was established to confirm that the laboratories were able to identify non-archetypal genotypes by MS typing. The panel consisted of DNAs from non-archetypal *T. gondii* strains (*n* = 7) and two archetypal strains (*n* = 2), provided in duplicate (Table 2). Concentrations of DNA were similar to those of the 10^−1^ dilution of the first part.

For each part, *T. gondii* DNAs were diluted in bovine carrier DNA with a concentration of 100 ng/μL. Two samples (first part) or one sample (second and third parts) of bovine carrier DNA alone was included as negative controls. The trial was blinded for all participants, including the organizing laboratory (E); only in the third part, the operators knew about the non-archetypal nature of some of the isolates included, but were unaware of the identity and order of the samples.

Irrespective of laboratory-specific protocols, each laboratory was asked to use 5 μL template DNA per reaction in the multiplex typing PCR.

After completing each part, interlaboratory divergences were assessed and discussed among the participants. All laboratories tried to improve their protocols and procedures for the subsequent part. The aim was to improve the individual typing results of each of the participating laboratories and to harmonize MS typing by using and extending the internal guidelines of laboratory B.

### Questionnaire to asses divergence from original method

A questionnaire was distributed to collate the technical and methodological details in each laboratory. During online meetings, further details on individual protocols, such as use of particular rules to analyze sequencing profiles, were recorded.

### Statistics

After reception of the data, all results were computed in tables for each part and studied separately (Supplementary File Table 1). For each sample, the coded number of the organizing laboratory and of the external laboratory was registered in an EXCEL file along with the operator, the software used, the typing results, the Ct value obtained by initial real-time PCR, the sample volume used for the reactions, the identified genetic type, and the number of typing markers identified. To compare the results among laboratories, the R software (R version 4.1.2, https://cran.r-project.org/) was used for linear regression and specifically, the R packages “binom,” “ggpubr,” “ggplot2,” and “cowplot” for calculating confidence intervals and preparing graphical representations of the results.

## Results

### Questionnaire results

All participants used the MS typing technique as reported previously [11]. However, questionnaire data and subsequent communication during online meetings revealed a number of deviations from the original protocol, even in the laboratory where the method had been initially established (laboratory B). Interestingly, one of the laboratories (A) had replaced the fluorophore HEX_Fl_ with VIC_Fl_, another (D) had replaced NED_Fl_ with TAMRA_Fl_, and two laboratories (B, E) had replaced NED_Fl_ with Atto550_Fl_, for three or two of the MS marker regions, respectively (Table 3).

**Table 3:** Sets of fluorophores used by the laboratories (A-E) participating in a ring trial on microsatellite typing of *Toxoplasma gondii*

**Group of markers**	**Laboratory**				
	**A**	**B**	**C**	**D**	**E**
**N61, B18, M33, M48, TUB2, N83, XI.1**	6-FAM_Fl_	6-FAM_Fl_	6-FAM_Fl_	6-FAM_Fl_	6-FAM_Fl_
**N82, TgM-A, W35, IV.1, B17**	VIC_Fl_	HEX_Fl_	HEX_Fl_	HEX_Fl_	HEX_Fl_
**N60, M102**	NED_Fl_	Atto550_Fl_	NED_Fl_	TAMRA_Fl_	Atto550_Fl_
**AA**	NED_Fl_	NED_Fl_	NED_Fl_	TAMRA_Fl_	Atto550_Fl_

Further differences were related to the types of sequencing devices, the size standards, and the software used to assess the fragment length of amplified MS regions (Table 4).

**Table 4:** Technical or methodological details of the microsatellite typing technique as retrieved by questionnaire and further personal communications from laboratories participating in a ring trial on microsatellite typing of *Toxoplasma gondii*

**Details**	**Laboratory, operator, or software**					
	**A-1**^**a**^	**A-2**^**a**^	**B**	**C**	**D**	**E-1 or E-2**^**b**^
Real-time PCR protocol used to quantify DNA	Adapted from [25]	Adapted from [25]	[26, 27]	[28]	[29]	Adapted from [30]
Target of real-time PCR used to quantify DNA	REP529, GenBank accession no. AF146527	REP529, GenBank accession no. AF146527	REP529, GenBank accession no. AF146527	REP529, GenBank accession no. AF146527	REP529, GenBank accession no. AF146527	REP529, GenBank accession no. AF146527
Template volumes used in real-time PCR	5 μL	5 μL	5 μL	5 μL	3 μL	10 μL
PCR MS typing (Part I)	15 MS multiplex	15 MS multiplex	15 MS multiplex + singleplexes	15 MS multiplex	8 MS multiplex (typing markers) + 7 MS multiplex (fingerprinting)	15 MS multiplex
Supplier/provider of typing primers	IN	IN	AB	AB	EU	ME (EU in comparative experiments)
Template volume used for MS multiplex PCR	5 μL	5 μL	5 μL	5 μL	5 μL	5 μL
Volume of amplification product used for fragment length analysis	1 μL	1 μL	1 μL	1 μL	1 μL	1 μL
Multiplex PCR kit used for typing	QIAGEN Multiplex PCR Kit (cat. no. 206143)	QIAGEN Multiplex PCR Kit (cat. no. 206143)	QIAGEN Multiplex PCR Kit (cat. no. 206143)	QIAGEN Multiplex PCR Kit (cat. no. 206143)	QIAGEN Multiplex PCR Kit (cat. no. 206143)	QIAGEN Multiplex PCR Kit (cat. no. 206143)
Capillary sequencer	3730 DNA Analyzer (ABI)	3730 DNA Analyzer (ABI)	3130xl Genetic Analyzer (ABI)	SeqStudio Genetic Analyzer (ABI)	3730 Genetic Analyzer (ABI)	Hitachi 3500 Genetic Analyzer (ABI)
Size standard	LIZ 500 (TF)	LIZ 500 (TF)	ROX 500 (ABI)	ROX 500 (ABI)	LIZ 500 (ABI)	ROX 500 (ABI)
MS typing software	Peak Scanner 2.0 (no bins established for allele identification)	Osiris (first part of ring trial only, no bins established for allele identification)	Gene-Mapper (bins established for allele identification)	Peak Scanner online (semi-automatic MS EXCEL template to ease allele identification)	Geneious Prime (bins established for allele identification)	Geneious Prime (bins established for allele identification)

^a^In laboratory A, two different software tools were used, i.e., Peak Scanner 2.0 (A-1) or Osiris (A-2). ^b^In laboratory E, two operators assessed raw data, i.e., electrophoresis profiles

### PCR results to quantify *T. gondii* DNA in samples

**Fig. 1:**
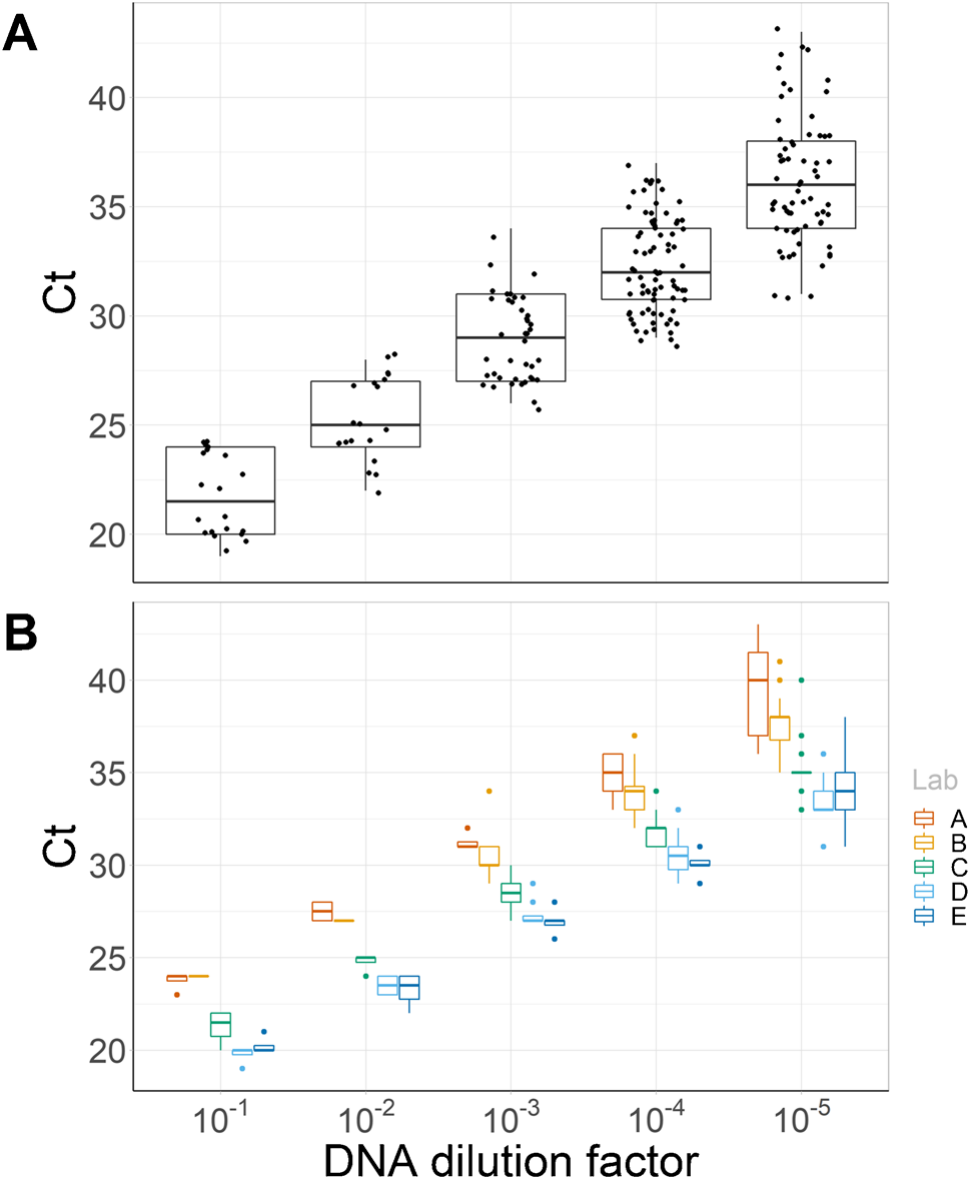
Real-time PCR results to quantify specific DNA in the samples of the first part of the ring trial on *Toxoplasma gondii* microsatellite typing according to the dilution of samples. **A** Median, 25–75% quantile (box), minimum and maximum (whiskers) of Ct values reported by all participants of the ring trial. **B** Median, 25–75% quantile (box), minimum and maximum (whiskers) of Ct values stratified for laboratories participating in the ring trial (Lab)

In the first part, linear regression analysis of the Ct values reported by each laboratory on serially diluted DNAs of reference isolates revealed *R*^2^ values between 0.577 and 0.710 for individual laboratories (Fig. 1; Table 5). Comparison of the individual regression line equations revealed that the Ct values reported by the laboratories differed. Laboratories D and E reported the lowest Ct values and laboratories A and B the highest for the same given strain (Table 5). Overall, the pairwise comparisons between Ct values reported by the different laboratories revealed *R*^2^ values between 0.94 and 0.84. The proportions of recognized positive samples ranged from 85.4% (laboratory D) to 100% (laboratory B) (Table 5). All laboratories reported negative results for negative control samples.

**Table 5:** Summarized results of the linear correlation between Ct value and DNA concentration in the samples of the first part of the ring trial on microsatellite typing for *Toxoplasma gondii*

**Laboratory**	***R***^**2**^	**Regression line equation**	**Proportion of positive samples recognized**
A	0.606	*y*= − 1.142ln(*x*) + 21.554	97.9%
B	0.653	*y*= − 1.035ln(*x*) + 21.818	100%
C	0.710	*y*= − 1.075ln(*x*) + 19.083	95.8%
D	0.577	*y*= − 0.974ln(*x*) + 18.629	85.4%
E	0.596	*y*= − 1.01ln(*x*) + 18.196	93.8%

### Typing archetypal *T. gondii* and impact of DNA concentration

The identification of the genetic type of samples was based on lineage typing markers (TUB2, W35, TgM-A, B18, B17, M33, IV.1, and XI.1). The proportion of correct identifications of the canonical types I, II, and III and a type II × III recombinant decreased depending on the dilution of the samples. If a participant had added a question mark to the result or provided an ambiguous typing result, NA was recorded. There were no differences in the typing results between the two software tools used by laboratory A and the two operators from laboratory E.

**Fig. 2:**
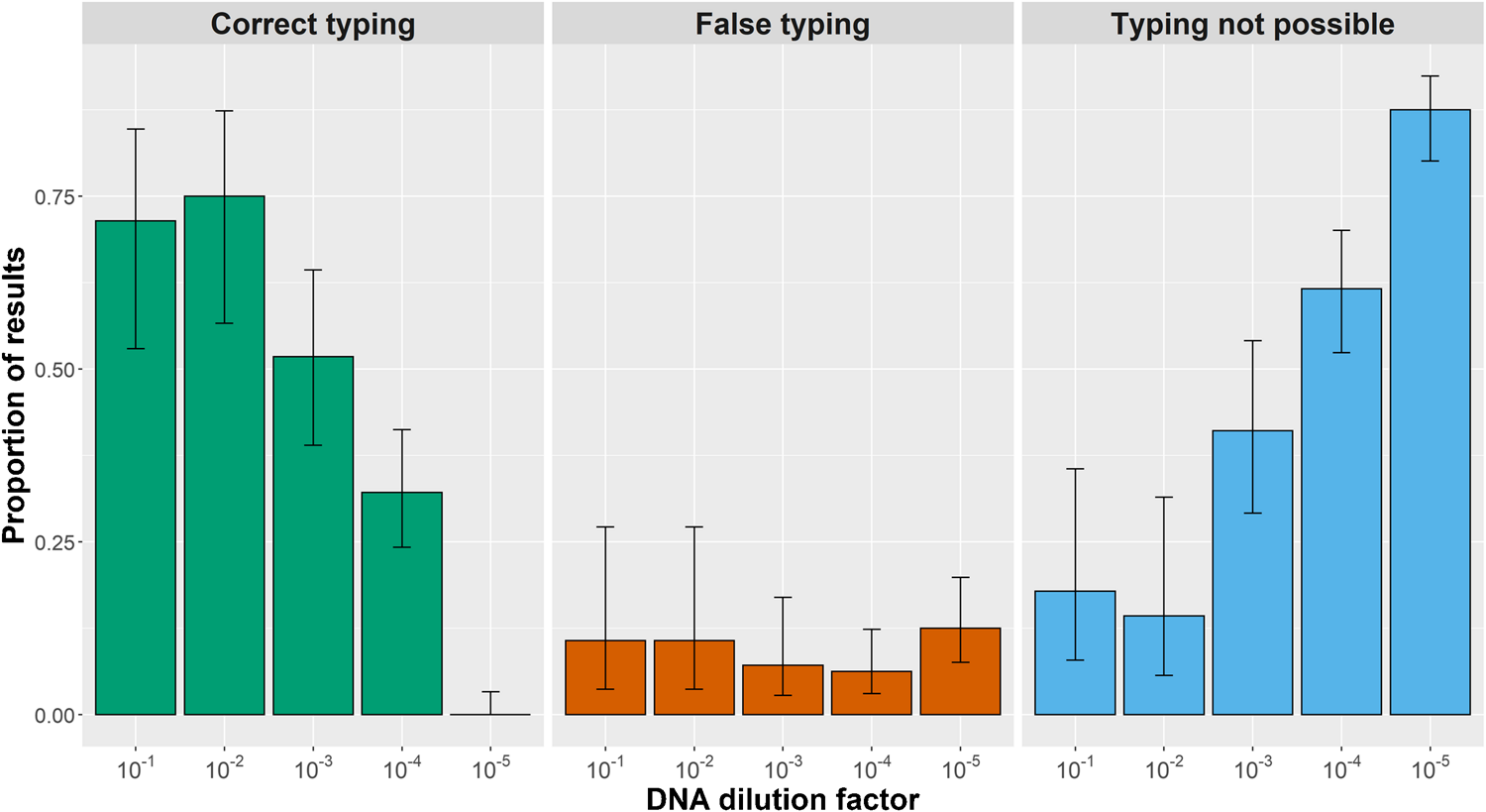
Relationship between typing results and DNA concentration: Proportion and 95% confidence intervals of correct (green) and false (orange) typing or typing not possible (blue) in the samples of the first part of the ring trial on *Toxoplasma gondii* microsatellite typing according to the dilution of samples for all laboratories and operators (i.e., type I, II, III, or II × III recombinant). Note: Number of replicas per strain DNA varied according to sample dilution (please refer to Table 1)

At the highest DNA concentrations, the 1st and 2nd dilutions, 71% (20/28) and 75% (21/28) of the results provided by all participants were correct. At the two following concentrations (3rd and 4th dilution), 52% (29/56) and 32% (36/112) of the typing outcome reported were correct. In contrast, the 5th dilution analysis revealed no (0/112) correct identification (Fig. 2). Overall, not only the proportion of incorrect typing results increased with higher dilution of the *T. gondii* DNA, but also the proportion of undetermined types, i.e., from 18% (5/28) or 14% (4/28) for the 1st and 2nd dilution to 41% (23/56), 62% (69/112), or 88% (98/112) for the 3rd, 4th, and 5th dilutions, respectively (Fig. 2).

If only the results for dilutions 1, 2, and 3 were included, small differences of up to minimum or maximum deviations of 2 bp relative to the results provided by the reference laboratory (B) were often recorded. In 27 cases, minimum or maximum values exceeding 2 bp were observed (Table 6). Most (63%; 17/27) deviations occurred in results reported by laboratory A. Some of the deviations were extreme and ranged up to 28 or 30 bp (laboratories A and C; Table 6).

**Table 6:** Results of the first part of the ring trial on microsatellite typing for *Toxoplasma gondii* in relation to results provided by laboratory B as a reference: Median [minimum; maximum] differences in length observed for each marker compared to the results provided by the reference laboratory (laboratory B). Median differences exceeding 1 bp are typed in bold. Minimum and maximum values exceeding 2 bp are underlined and in italics. Note: The use of Atto550 for N60 and M102 by reference laboratory B was a deviation from the original method. The analysis was restricted to sample dilutions 1, 2, and 3

**Marker**	**Median differences [minimum; maximum] in fragment size relative to the results of laboratory B stratified by laboratory operator or software**					
	A-1^a^	A-2^a^	**C**	**D**	**E-1**^**b**^	**E-2**^**b**^
Lineage typing marker						
B18	0 [− 1;0]	0 [− 1;0]	0 [0;4]	0 [0;0]	0 [0;0]	0 [0;0]
M33	0 [0;2]	0 [0;2]	1 [0;1]	0 [0;0]	0 [0;0]	0 [0;0]
TUB2	0 [0;0]	0 [0;0]	0 [0;0]	0 [0;0]	0 [0;0]	0 [0;0]
XI.1	0 [0;1]	0 [0;1]	0 [0;1]	0 [0;0]	0 [0;0]	0 [0;0]
TgM-A	0 [0;1]^c^	0 [− *12*;1]^c^	0 [− 1;0]	0 [0;0]	0 [0;0]	0 [0;0]
W35	**3** [0;*3*]^c^	**3** [0;*3*]^c^	0 [0;0]	0 [0;0]	0 [0;0]	0 [0;0]
IV.1	**2** [− *4*;2]^c^	**2** [− *4*;2]^c^	0 [0;1]	0 [0;2]	0 [0;0]	0 [0;0]
B17	0 [− *4*;*28*]^c^	0 [− 1;*28*]^c^	0 [0;1]	0 [− 2;0]	0 [0;0]	0 [0;0]
Fingerprinting marker						
N82	**2** [0;2]^c^	**2** [1;*9*]^a^	**2** [0;*15*]	0 [0;2]	0 [0;0]	0 [0;0]
N61	**4** [1;*10*]	**3** [1;*10*]	0 [0;*30*]	0 [**−** 2;0]	0 [0;0]	0 [0;0]
M48	1 [*− 3*;2]	1 [**−** 2;2]	1 [− 3;3]	0 [0;2]	0 [0;0]	0 [0;0]
N83	− 1 [− 1;0]	**−** 1 [**−** 1;0]	0 [0;2]	0 [0;0]	0 [0;0]	0 [0;0]
N60	**− 5** [*− 5*;0]^d^	**− 4** [*− 5*;0]^d^	**− 5** [*− 5*;0]^d^	1 [**−** 1;1]^e^	1 [1;1]	1 [1;1]
M102	**− 2** [*− 7*;0]^d^	**− 2** [*− 7*;0]^d^	**− 2** [*− 3*;0]^d^	**2** [*− 4*;*4*]^e^	**2** [0;2]	**2** [0;2]
AA	**2** [0;*5*]	**2** [0;*5*]	**2** [0;2]	1 [0;*3*]^f^	**3.5** [*3*;*5*]^g^	**3.5** [*3*;*5*]^g^

^a^In laboratory A, two different software tools were used, i.e., Peak Scanner 2.0 (A-1) or Osiris (A-2). ^b^In laboratory E, two operators (E-1, E-2) assessed raw data, i.e., electrophoresis profiles; Differences to reference laboratory: ^c^VIC_Fl_ instead of HEX_Fl_; ^d^NED_Fl_ instead of Atto550_Fl_; ^e^TAMRA_Fl_ instead Atto550; ^f^TAMRA_Fl_ instead of NED_Fl_; ^g^Atto550_Fl_ instead of NED_Fl_

Median differences in the typing results relative to reference laboratory B were not equally distributed among the laboratories. Overall, the majority (70%; 16/23) of the major differences (i.e., median differences > 1 bp) occurred in particular markers, for which participants employed primers labeled with different fluorophores compared to reference laboratory B. In lineage typing, major differences were only observed between laboratory A and the reference laboratory (100%; 4/4). In the affected marker regions, laboratory A had used VIC_Fl_ instead of HEX_Fl_ to label fragments (Table 6). In fingerprinting analysis, all laboratories reported differences of > 1 bp relative to laboratory B. Of 20 differences, 12 occurred in cases with differences in labeling (Table 6). If only median differences of > 2 bp were counted, 78% (7/9) of the differences occurred in cases with differences in fluorophore labeling (NED_Fl_ instead of Atto550_Fl_ and vice versa, and TAMRA_Fl_ instead of NED_Fl_; details on primer labeling in Table 3).

Based on the results of the first part of the ring trial, it was observed that the fluorophore attached to primers for amplification of MS markers may have had an impact on the fragment sizes determined by capillary sequencing (Table 7). Thus, the literature on this topic was reviewed and differences in publications on the MS typing of RH, ME49, and NED strains were observed on 20 occasions (Table 7). In the vast majority of these cases (*n* = 17), the laboratory had used an alternative to the originally reported fluorophore, i.e., HEX_Fl_ was replaced with VIC_Fl_, NED_Fl_ with Atto550_Fl_, or NED_Fl_ with TAMRA_Fl_ (Table 7). Also, the reference laboratory B had recently started to replace NED_Fl_ with Atto550_Fl_ for the amplification of the N60_Fl_ and M102_Fl_ markers (Table 7).

**Table 7:** Results of the first part of the ring trial on microsatellite typing for *Toxoplasma gondii* in relation to literature data: Median differences in length observed for each marker of reference strains compared to the results provided in literature, i.e., for RH [31] or ME49 and NED [32]. Only markers are listed, for which laboratories used other fluorophores than those reported in the original reference. In the case of median differences exceeding 1 bp, entries are typed in bold. Fluorophores, different from the original description, are also indicated in bold. The analysis was restricted to sample dilutions 1, 2, and 3

**Marker**	**Median differences in fragment size relative to literature, number of observations, fluorophore stratified by laboratory operator or software**						
	**A-1**^**a**^	**A-2**^**a**^	**B**	**C**	**D**	**E-1**^**b**^	**E-2**^**b**^
N82	**1.6**, *n*=12, **VIC**_Fl_	**1.6**, *n*=11, **VIC**_Fl_	0, *n*=12, HEX_Fl_	**2**, *n*=10, HEX_Fl_	0, *n*=11, HEX_Fl_	0, *n*=12, HEX_Fl_	0, *n*=12, HEX_Fl_
TgM-A	0.4, *n*=11, **VIC**_Fl_	0.5, *n*=10, **VIC**_Fl_	0, *n*=12, HEX_Fl_	0, *n*=10, HEX_Fl_	0, *n*=11, HEX_Fl_	0, *n*=12, HEX_Fl_	0, *n*=12, HEX_Fl_
W35	**2.9**, *n*=9, **VIC**_Fl_	**2.8**, *n*=9, **VIC**_Fl_	0, *n*=9, HEX_Fl_	0, *n*=8, HEX_Fl_	0, *n*=11, HEX_Fl_	0, *n*=12, HEX_Fl_	0, *n*=12, HEX_Fl_
IV.1	**2,** *n*=9, **VIC**_Fl_	**2**, *n*=10, **VIC**_Fl_	0, *n*=12, HEX_Fl_	0, *n*=7, HEX_Fl_	0, *n*=11, HEX_Fl_	0, *n*=12, HEX_Fl_	0, *n*=12, HEX_Fl_
B17	− 0.3, *n*=9, **VIC**_Fl_	− 0.35, *n*=10, **VIC**_Fl_	0, *n*=12, HEX_Fl_	0, *n*=8, HEX_Fl_	0, *n*=11, HEX_Fl_	0, *n*=12, HEX_Fl_	0, *n*=12, HEX_Fl_
N60	− 0.2, *n*=11 NED_Fl_	− 0.3, *n*=11, NED_Fl_	**4**, *n*=12, **Atto550**_Fl_	− 1, *n*=10, NED_Fl_	**5**, *n*=12, **TAMRA**_Fl_	**5**, *n*=12, **Atto550**_Fl_	**5**, *n*=12, **Atto550**_Fl_
M102	− 0.5, *n*=9, NED_Fl_	− 0.3, *n*=9, NED_Fl_	**2**, *n*=11, **Atto550**_Fl_	0, *n*=9, NED_Fl_	**4**, *n*=12, **TAMRA**_Fl_	**4**, *n*=12, **Atto550**_Fl_	**4**, *n*=12, **Atto550**_Fl_
AA	**1.8**, *n*=11, NED_Fl_	**1.6**, *n*=11, NED_Fl_	0, *n*=11, NED_Fl_	**2**, *n*=7, NED_Fl_	**3**, *n*=12, **TAMRA**_**Fl**_	**3**, *n*=12, **Atto550**_Fl_	**3**, *n*=12, **Atto550**_Fl_

^a^In laboratory A, two different software tools were used, i.e., Peak Scanner 2.0 (A-1) or Osiris (A-2). ^b^In laboratory E, two operators (E-1, E-2) assessed raw data, i.e., electrophoresis profiles

### Fingerprinting *T. gondii* type II

#### Divergences in fragment length determination

Fingerprinting markers (M48, M102, N60, N82, AA, N61, and N83) can be used to differentiate strains within the same lineage. In the second part of the ring trial, laboratories B and E reported identical fingerprinting results in all regions of the duplicates of 10 different strains, i.e., on 70 occasions (10 strains, seven fingerprinting regions). While laboratory A reported non-existing differences in two (3%), laboratories C and D reported non-existing differences in seven (10%) or 18 (25%) of 70 occasions, respectively (Table 8).

**Table 8:** Failure in identifying duplicates in samples of the second part of the ring trial on microsatellite typing for *Toxoplasma gondii* per laboratory

**DNA number of providing laboratory**	**Success in identifying duplicates stratified by laboratory operator or software**				
	**A-1**^**a**^	**B**	**C**	**D**	**E-1 or E-2**^**b**^
D200109	AA‾	-	N61‾	AA^□^, N83^□^	-
D200111	-	-	-	AA^□^, N83^□^	-
D200113	-	-	N60^□^, N61‾	M48^□^, N61^□^, N82^□^	-
D200114	-	-	-	N82^□^, AA^□^, N83^□^	-
D200118	-	-	N60‾, N61^□^	M48^□^, AA^□^, N83^□^	-
D200119	AA^□^	-	N61‾	N61^□^	-
D200121	-	-	-	N82^□^	-
D200127	-	-	N61‾	-	-
D200129	-	-	-	N82^□^	-
D212556	-	-	-	M48^□^, N82^□^	-
Negative control	-	-	-	-	-

^a^In laboratory A, Peak Scanner 2.0 (A-1) was used as software tool. ^b^In laboratory E, two operators (E-1, E-2) assessed raw data, i.e., electrophoresis profiles; ‾: 1 bp difference; ^□^: 2 bp difference

#### Interlaboratory divergence in fragment length determination

In the second part of the ring trial, most of the differences recorded relative to reference laboratory B did not exceed minimum or maximum deviations of >2 bp. Unlike in the first part of the ring trial, minimum or maximum values exceeding 2 bp were observed in less cases (*n* = 10; Table 9). Most (60%; 6/10) of these deviations occurred in results reported by laboratory A. However, deviations were far less extreme as compared to the first part and ranged up to 6 bp in one laboratory (laboratory E; Table 9).

**Table 9:** Second part of the ring trial on microsatellite typing for *Toxoplasma gondii*: Median [minimum; maximum] differences in length observed for each marker compared to the reference laboratory. Median differences exceeding 1 bp are typed in bold. Minimum and maximum values exceeding 2 bp are underlined and in italics. Note: The use of Atto550_Fl_ for N60 and M102 by reference laboratory B was a deviation from the original method

**Marker**	**Median differences [minimum; maximum] in fragment size relative to results of laboratory B stratified by laboratory operator or software**				
	**A-1**^**a**^	**C**	**D**	**E-1**^**b**^	**E-2**^**b**^
Lineage typing marker					
B18	0 [0;0]	0 [0;0]	0 [0;0]	0 [0;0]	0 [0;0]
M33	0 [− 1;0]	0 [0;0]	0 [0;0]	0 [0;0]	0 [0;0]
TUB2	1 [0;1]	0 [0;0]	0 [0;0]	0 [0;0]	0 [0;0]
XI.1	1 [0;1]	0 [0;1]	0 [0;0]	0 [0;0]	0 [0;0]
TgM-A	1 [1;1]	0 [0;0]	0 [0;0]	0 [0;0]	0 [0;0]
W35	**3** [*3;3*]^c^	0 [0;1]	0 [0;0]	0 [0;0]	0 [0;0]
IV.1	**2** [2;2]^c^	0 [0;1]	0 [0;0]	0 [0;0]	0 [0;0]
B17	0 [0;0]^c^	0 [0;1]	0 [0;0]	0 [0;0]	0 [0;0]
Fingerprinting marker					
N82	**1.5** [1;2]^c^	**2** [2;2]	**2** [0;2]	0 [0;0]	0 [0;0]
N61	1 [1;1]	1 [0;2]	0 [− 2;2]	0 [0;0]	0 [0;0]
M48	**2** [2;2]	1 [1;2]	**2** [0;2]	0 [− 1;1]	0 [− 1;1]
N83	0 [− 1;0]	0 [0;0]	0 [− 2;0]	0 [0;0]	0 [0;0]
N60	− **5** [−﻿ ﻿*﻿﻿5;*− 3]^d^	− **5** [− 5*;*− 3]^d^	− 1 [− 1;0]^e^	1 [1;2]	0 [0;2]
M102	− **3** [− ﻿*﻿﻿3;*− 3]^d^	− **2** [− 2; − 2]^d^	**2** [2;2]^e^	**2** [2;2]	**2** [0;2]
AA	**2** [− 1;2]	**2** [1;3]	**2** [0;2]^f^	**3** [*3;5*]^g^	**4** [*4;6*]^g^

^a^In laboratory A, Peak Scanner 2.0 (A-1) was used as software tool. ^b^In laboratory E, two operators (E-1, E-2) assessed raw data, i.e., electrophoresis profiles; Differences to reference laboratory: ^c^VIC_Fl_ instead of HEX_Fl_; ^d^NED_Fl_ instead of Atto550_Fl_; ^e^TAMRA_Fl_ instead Atto550; ^f^TAMRA_Fl_ instead of NED_Fl_; ^g^Atto550_Fl_ instead of NED_Fl_

### Typing non-archetypal *T. gondii* strains

#### Divergence in fragment length determination in duplicated samples

In the third part, not only fingerprinting, but also typing markers varied between the isolates. Since the samples had been provided in duplicate, it was possible to assess the extent, to which duplicates were correctly recognized. Compared to the second part of the ring trial, the ability to recognize samples with identical profiles increased for all laboratories except laboratory C (i.e., *n* = 7 in second part but *n* = 10 in third part). The results for one marker (IV.1) were not available for analysis in the case of one isolate (FRENCHGUIANA15) in laboratories A, C, and E, because they failed to amplify this marker (Table 10).

**Table 10:** Failure in identifying duplicates in samples of the third part of the ring trial on microsatellite typing for *Toxoplasma gondii* per laboratory

**DNA designation of providing laboratory**	**Success in identifying duplicates stratified by laboratory operator or software**				
	**A-1**^**a**^	**B**	**C**	**D**	**E-1**^**b**^
BENIN02	-	-	N61‾, N83^□^	-	-
FRENCHGUIANA01	-	-	-	-	-
GABON02	-	-	XI.1‾	-	-
GUADELOUPE02	-	-	-	-	-
MARTINIQUE03	-	-	-	-	-
SENEGAL17	-	-	W35‾, IV.1‾, N61‾	-	-
GABON03	-	-	IV.1‾, N61^□^	-	-
FRENCHGUIANA11	-	-	IV.1‾	-	-
FRENCHGUIANA15	IV.1^NA^	-	N61‾, IV.1^NA^	M102^□^, N82^□^	IV.1^NA^
Negative control	-	-	-	-	-

^a^In laboratory A, Peak Scanner 2.0 (A-1) was used as software tool. ^b^In laboratory E, the operator E-1 assessed raw data, i.e., electrophoresis profiles; ‾: 1 bp difference; ^□^: 2 bp difference; NA: no result for IV.1

#### Interlaboratory divergence in fragment length determination

In the third part, most of the differences recorded relative to reference laboratory B did not exceed minimum or maximum deviations of > 2 bp. Compared to the other parts of the ring trial, minimum or maximum values exceeding 2 bp were observed in a small number of cases (*n* = 7; Table 11). The majority (71%; 5/7) of such deviations occurred in the results of laboratories that had chosen fluorophores that differed from those used by the reference laboratory (Table 11).

**Table 11:** Third part of the ring trial on microsatellite typing for *Toxoplasma gondii*: Median [minimum; maximum] differences in length observed for each marker compared to the results reported by the reference laboratory. Median differences exceeding 1 bp are typed in bold. Minimum and maximum values exceeding 2 bp are underlined and in italics. Note: The use of Atto550_Fl_ for N60_Fl_ and M102_Fl_ by reference laboratory B was a deviation from the original method

**Marker**	**Median [minimum; maximum] differences per laboratory, software or operator**			
	**A-1**^**a**^	**C**	**D**	**E-1**^**b**^
Lineage typing marker				
B18	0 [0;0]	0 [0;0]	0 [0;0]	0 [0;0]
M33	0 [0;0]	0 [0;0]	0 [− 2;0]	0 [− 2;0]
TUB2	0 [0;0]	0 [− 2;0]	0 [0;0]	0 [0;0]
XI.1	0 [0;0]	0 [0;0.5]	0 [0;0]	0 [0;0]
TgM-A	0 [0;0]	0 [0;1]	0 [0;0]	0 [0;0]
W35	0 [0;0]^c^	0 [0;0.5]	0 [0;0]	0 [0;0]
IV.1	0 [0;0]^c^	0 [0;0.5]	0 [0;0]	0 [0;0]
B17	0 [− 2;0]^c^	0 [− 1;0]	− **2** [− 2; − 2]	0 [− 2;0]
Fingerprinting marker				
N82	**2** [2;2]^c^	**2** [2;2]	**2** [1;2]	0 [0;0]
N61	0 [0;0]	0.5 [0;1.5]	2 [2;2]	0 [0;0]
M48	0 [0;0]	1 [1;2]	0 [0;0]	0 [0;0]
N83	0 [0;0]	0 [0;1]	0 [0;0]	0 [0;0]
N60	− **4** [− ﻿*﻿5;*− 4]^d^	− **4** [− 5*;*− 4]^d^	− **2** [− 2;− 3]^e^	**2** [2;*3*]
M102	− **4** [− ﻿*﻿4;*− 4]^d^	− **2** [− 2;− 1]^d^	− 0 [− 1;0]^e^	**2** [2;2]
AA	0 [0;0]	**2** [2;*3*]	2 [2;2]^f^	**4** [4*;*4]^g^

^a^In laboratory A, Peak Scanner 2.0 (A-1) was used as software tool. ^b^In laboratory E, the operator E-1 assessed raw data, i.e., electrophoresis profiles; Differences to reference laboratory: ^c^VIC_Fl_ instead of HEX_Fl_; ^d^NED_Fl_ instead of Atto550_Fl_; ^e^TAMRA_Fl_ instead Atto550_Fl_; ^f^TAMRA_Fl_ instead of NED_Fl_; ^g^Atto550_Fl_ instead of NED_Fl_

All laboratories correctly identified archetypal *T. gondii* type III or type II variants, although laboratories C and D did not report the variation in this isolate (Table 12). All laboratories, except laboratory C, recognized all non-archetypal strains as such. Laboratory C misclassified Africa 1 as type I, and the Caribbean 1, 2, and 3 as type III, and for two Amazonian isolates the result “Unclassified” was provided. All remaining laboratories, except laboratory C, correctly identified Caribbean 1, 2, and 3, determined the Amazonian isolate as Unclassified or as Amazonian, and the type III-like isolate as Unclassified (laboratory D), type III variant (laboratories B and E), or South American 4-like (laboratory A).

**Table 12:** Typing results reported by the laboratories (A–E) compared to results reported in the literature: The set provided in the third part of the ring trial on microsatellite typing for *Toxoplasma gondii* comprised of two strains with an archetypal and seven strains with non-archetypal genotype

**Sample ID**	**Type according to literature**	**Type determined by laboratory**				
		**A-1**^**a**^	**B**	**C**	**D**	**E-1**^**b**^
BENIN02	Africa 1	Africa 1	Africa 1	I	Africa 1	Africa 1
GABON03	III	III	III	III	III	III
GABON02	III-like	South American 4-like	III variant TUB2	III	Unclassified	III variant TUB2
FRENCHGUIANA15	Amazonian	Unclassified	Amazonian	Unclassified	Amazonian	Unclassified
FRENCHGUIANA11	Amazonian	Unclassified	Amazonian	Unclassified	Amazonian	Unclassified
GUADELOUPE02	Caribbean 2	Caribbean 2	Caribbean 2	III	Caribbean 2	Caribbean 2
FRENCHGUIANA01	Caribbean 1	Caribbean 1	Caribbean 1	III	Caribbean 1	Caribbean 1
MARTINIQUE03	Caribbean 3	Caribbean 3	Caribbean 3	III	Caribbean 3	Caribbean 3
SENEGAL17	II^c^	II-like	II variant W35	II	II	II variant W35

^a^In laboratory A, Peak Scanner 2.0 (A-1) was used as software tool. ^b^In laboratory E, the operator E-1 assessed raw data, i.e., electrophoresis profiles. ^c^Designated as type II in literature although W35 showed a variation from type II pattern (244 bp instead of 242)

### Effects due to use of different fluorophore labeling and different suppliers for primers

To confirm that the different fluorophores used caused differences in the apparent sizes of amplified PCR products, comparative experiments were performed using the DNAs from RH, ME49, and NED reference strains and the primer pairs corresponding to the marker regions N60, M102, and AA provided by the different laboratories, labeled with NED_Fl_, TAMRA_Fl_ or Atto550_Fl_. Capillary sequencing as well as the assessment of profiles was done in laboratory E. The results obtained in laboratory E using reagents provided by reference laboratory B were identical with those previously obtained by laboratory B.

### Different fluorophore labeling

NED_Fl_-labeled N60 fragments were 4 to 5 bp shorter and M102 fragments 2 bp shorter as compared to the Atto550_Fl_-labeled reference (Table 13). TAMRA_Fl_-labeled N60 was 2 bp shorter compared to Atto550_Fl_-labeled reference, while the M102 fragments had the same length.

**Table 13:** Effect of different fluorophores and primer suppliers on microsatellite fragment sizes: Differences in microsatellite (MS) typing for *Toxoplasma gondii* between Atto550_Fl_- and NED_Fl_-labeled MS fragments N60, M102, and AA for reference *T. gondii* strains RH, ME49, and NED using reagents provided to laboratory E by laboratories participating of the ring trial on *T. gondii* microsatellite typing

**Marker**	**Strain**	**Laboratory, commercial supplier of primer, primer labeling**						
		**Fragment size determined with reagents provided by reference laboratory B: supplier, fluorophore**		**Differences between fragment size determined in laboratory E using reagents of participating laboratories: laboratory, supplier, fluorophore**				
		**AB, Atto550**_**Fl**_	**AB, NED**_**Fl**_	**A, IN, NED**_**Fl**_	**C, AB, NED**_**Fl**_	**D, EU, TAMRA**_**Fl**_	**E, ME, Atto550**_**Fl**_	**E, EU, Atto550**_**Fl**_
N60	RH	149	NA	− 4	− 4	− 2	2	0
	ME49	147	NA	− 5	− 5	− 2	2	0
	NED	151	NA	− 4	− 4	− 2	2	0
M102	RH	168	NA	− 2	− 2	0	2	0
	ME49	176	NA	− 2	− 2	0	2	0
	NED	192	NA	− 2	− 2	0	2	0
AA	RH	NA	265	0	0	2	4	2
	ME49	NA	265	0	0	2	4	2
	NED	NA	267	0	0	2	4	2

### Different suppliers for primers

The Atto550_Fl_-labeled N60 fragments were identical to the reference Atto550_Fl_-labeled fragments, if primer pairs supplied by company EU were used by laboratory E. In contrast, if Atto550_Fl_-labeled primer pairs bought from company ME were used by laboratory E, fragments were 2 bp longer than the reference Atto550_Fl_-labeled fragments (Table 13). In the case of the AA marker, TAMRA_Fl_ and Atto550_Fl_ (EU) fragments were 2 bp longer and Atto550_Fl_ (ME) fragments were 4 bp longer than reference NED_Fl_ fragments.

## Discussion

Typing of *T. gondii* strains is important to study the global population structure of the parasite*.* Genomic diversity of *T. gondii* may influence the epidemiology of the parasite, affecting, for example, definitive host and intermediate host adaptation [23, 33, 34]. In addition, some *T. gondii* genotypes are reported to have a higher virulence for particular hosts than other genotypes [35, 36]. Such differences in virulence may exist between different host species, but also at the intra-host-species level [37, 38].

Multilocus MS typing was established more than one decade ago [11] and has proven to be suitable to discriminate *T. gondii* strains at the level of lineages globally [8, 15, 39] as well as on the intra-lineage level [17, 40]. Essentially, laboratories currently use this technique to study strains and clinical samples from different geographical areas. As a consequence, differences in typing results between laboratories could introduce bias in population genetic studies comparing MS genotypes from different geographical locations. This is also true within the same geographical region such as in Europe with several laboratories using MS genotyping [9].

Our study revealed numerous differences in MS typing protocols, although all participants of the ring trial, including the reference laboratory, referred to the original description of the MS typing methodology [11]. Laboratories used different real-time PCR procedures to quantify *T. gondii* DNA prior to typing, different fluorophores (Table 3), capillary sequencers, size standards, and different software tools to assess fragment length of amplified marker regions (Table 4). Differences in the allele identification, supported by various software tools, were noted. Only with particular software, not available to all participants, was it possible to ease and automatize allele identification (Table 4). Not all participating laboratories used the same software and some were not able to automatize allele identification in the respective software. Some of the applied tools (i.e., Gene-Mapper and Geneious Prime) allowed the definition of loci and bins to ease allele identification. The inclusion of characterized reference DNAs can help to define loci and subsequently the respective bins. Furthermore, participating laboratories had different levels of experience in *T. gondii* MS typing, which ranged from several years to a few weeks.

The first part of this ring trial focused on lineage typing and the effect of *T. gondii* DNA concentrations on typing. As usually done for field samples, each laboratory tried to quantify *T. gondii* DNAs in the samples. Each laboratory used a different real-time PCR protocol (Table 4); however, overall high correlation coefficients between Ct values and DNA content in samples were observed. Nevertheless, Ct values differed by more than 3 Ct units in some cases (Table 5). This must be kept in mind for the interpretation of results reported in the literature. Nevertheless, laboratory-specific Ct values provide valuable information for optimizing DNA concentrations of samples (e.g., field samples) subsequently used for MS typing.

**Fig. 3:**
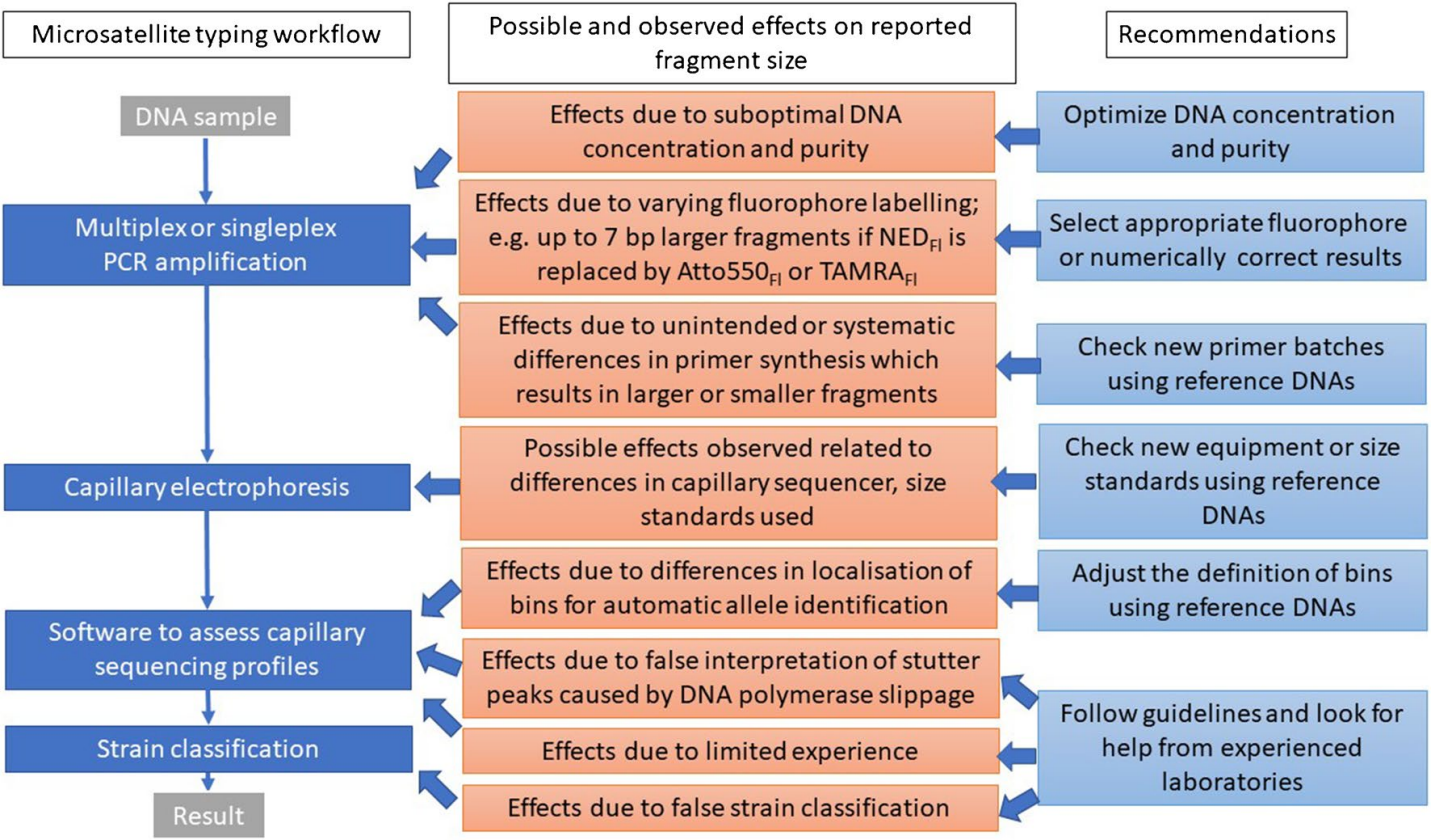
Possible and observed effects on the *Toxoplasma gondii* microsatellite marker fragment size determination: Steps affected in the microsatellite typing workflow and recommendations for optimization

The results revealed that from dilution 3 (0.01 ng/μL *T. gondii* DNA) onwards, the proportion of samples increased, in which laboratories were no longer able to determine the lineage type. However, the proportion of reporting incorrect lineage typing results did not increase with decreasing DNA content. The results were consistent among the participating laboratories and relative to the results of the reference laboratory only up to dilution 2 (0.1 ng/μL *T. gondii* DNA). Thus, it seems to be important to estimate the level of *T. gondii* DNA concentration in samples prior to MS typing, and to use this information to select those samples, for which lineage typing (and subtyping) is most likely possible, or to optimize DNA concentration for typing (Fig. 3).

It should be noted that not only a limited concentration of DNA may negatively influence the accuracy of determining the correct fragment size, but also an excess of *T. gondii* DNA may cause problems. In the first part of this ring trial, it was noted that the proportion of correct lineage typing was lower in dilution 1 (1 ng/μL *T. gondii* DNA) than in dilution 2 (0.1 ng/μL *T. gondii* DNA) samples. It has been noted previously that an overrepresentation of target DNA may cause so-called minus-A peaks during capillary electrophoresis [41]. Minus-A peaks can occur, if a number of amplified fragments lack a terminal adenine at the 3′ end, which is usually added by many DNA polymerases without the use of the template. We studied the occurrence of minus-A peaks for the markers M33 and M102. Both markers showed double peaks, where the intensity of the first peak (assumed to be a minus-A peak as detailed in the typing guidelines, provided as Supplementary File Text 1) increased with increasing DNA concentrations, while the intensity of the second peak (assumed to be the correct peak) decreased. This can cause incorrect results, if the operator or the software normally choose the highest peak as the correct one.

Major differences, mainly affecting fingerprinting markers, were observed in results reported in all parts of the ring trial, especially among those laboratories that used different fluorophores for labeling forward primers (Tables 6, 7, 9, and 11). Comparative experiments performed exclusively in laboratory E, but using primers from the other participating laboratories, confirmed these observations (Table 13). Effects on apparent fragment sizes in capillary sequencing due to differences in fluorophore labeling, especially for fluorescein and rhodamine dyes, have been reported previously [42]. However, these effects and their root-causes remained largely understudied. The fluorophores used in our study, Atto550_Fl_ and TAMRA_Fl_, are rhodamine dyes, while NED_Fl_ belongs to the fluorescein dyes. The previous study reported that TAMRA_Fl_-labeled fragments tended to be larger than NED_Fl_-labeled fragments and that this effect depended on the fragment size, i.e., the smaller the fragment, the stronger the retardation in capillary electrophoresis of TAMRA_Fl_ relative to NED_Fl_-labeled fragments [42]. Results of comparative experiments with various reagents in laboratory E (Table 13) were mainly in accord with this observation for markers labeled with NED_Fl_ in the original protocol. The strongest effects of a 4–5-bp retardation in Atto550_Fl_ relative to NED_Fl_-labeled fragments were observed in the smallest fragment N60 (149–151 bp) and a 2-bp retardation in the larger fragments M102 (168–192 bp) and AA (265–267 bp). TAMRA_Fl_-labeled fragments also appeared to be 2 bp larger relative to NED_Fl_-labeled fragments, but size-dependent differences could not be determined.

In contrast to TAMRA_Fl_ and Atto550_Fl_, VIC_Fl_ (used in laboratory A instead of HEX_Fl_) belongs to the fluorescein-like dyes, similar to HEX_Fl_, so no effects on fragment size were expected. This was confirmed in our analysis.

It should be mentioned here that an additional retardation of 2 bp was noted when Atto550_Fl_-labeled primer pairs, used to amplify N60, M102, or AA, had been purchased from the company ME and not from the companies AB or EU (Table 13). The reasons for the differences related to the primer supplier remain unknown. A potential error in the order of the primers was excluded and it should be noted that all primer pairs with different sequences from this supplier were affected. Most likely, the differences seem to be linked to primer production. Differences in the chemical reactions applied to label primers with fluorophores may be possible.

Thus, in general it seems to be important to validate new reagents by using defined reference DNAs, ideally included in each run of capillary sequencing (Fig. 3). In addition, comparative experiments with defined reference DNAs should become mandatory, if the method is newly established in a laboratory or even if previously used primers are replaced by new ones (Fig. 3). In our view, it is unlikely that different PCR kits or enzymes contribute to differences in the amplified fragments, but this was not assessed in our ring trial because all participants used the same multiplex PCR kit.

Results for MS typing were discussed among the participants in web-based meetings. Overall, an improvement of typing results relative to those of the reference laboratory was observed between the first and second parts of the ring trial, probably because participants gained experience and were given access to a laboratory internal guideline established in reference laboratory B. While one of the laboratories with little previous experience (laboratory A) obtained results that differed in determined fragment sizes relative to results provided by the reference laboratory by up to 28 bp (Table 6), including only dilution 1 and 2 results, this was no longer the case in further parts of the ring trial. In the second and third parts, deviations of a maximum of 5 bp were observed (Tables 9 and 11). This clearly shows the need for guidance, if *T. gondii* MS typing is newly established in a laboratory (Fig. 3).

So-called stutter peaks are frequently reported in MS typing (examples are displayed in the typing guidelines, provided as Supplementary File Text 1) and are caused by slippage of the DNA polymerase. They occur more often, when the number of MS repeats is >20, and less frequent, if the repeat number is <10 [41]. Specific guidelines (laboratory-specific guidelines similar to the guidelines provided in Supplementary File Text 1) may provide help to identify the correct fragment size (Fig. 3). However, stutter peaks remain a problem in MS typing, because it may not always be possible to determine the true variation in repeat numbers in the original DNA.

In the final part of the ring trial, DNA from strains not belonging to the archetypal lineages types I, II, and III was analyzed in the participating laboratories. Although the differences in the results were minor, especially for the typing markers (Table 12), none of the exotic strains was correctly classified by the semi-automatic system in place in one of the laboratories (laboratory C) (Table 2), due to limited references in the system version. Based on the results of laboratory C, correct typing would have been possible, if additional non-archetypal references would have been added to the system. This highlights the challenges of automatization for an organism with substantial genetic variation. One of the isolates was classified by some of the laboratories as type II, and by others as type II variant or type II-like, which shows that also for MS-based classification of lineages or the nomenclature of genotypes clear rules or guidelines are necessary (Fig. 3).

In conclusion, the results of this interlaboratory ring trial suggest that harmonization of MS typing appears to be possible, which might allow the combination of larger data sets on *T. gondii* genotypes. This is an important prerequisite to study and unravel the molecular epidemiology of this parasite. The use of different fluorophores to label fragments during amplification was identified as a major source of divergence. After numerical adjustments of fragment size results, based on comparative analyses using defined reference DNAs, differences due to the use of other fluorophores no longer presented a problem and results were comparable to those previously reported in the literature. In addition, minor differences of 2 bp could be attributed to different primer suppliers. Further minor differences probably resulted from limited experience, less suitable software for assessing capillary electrophoresis profiles, and missing software options to automatize allele identification. These observations are not only important for typing *T. gondii*, but may also be relevant for other applications of MS typing (i.e., forensic identification and relatedness testing, cell line identification, or population studies).

## Supplementary information

ESM 1 (PDF 1.44 MB)

ESM 2  (XLSX 87.2 KB)

## Data Availability

The datasets generated and/or analyzed during the current study are available as Supplementary File Data Table 1.
