# Supplementary material for: A ring trial to harmonize Toxoplasma gondii microsatellite typing: comparative analysis of results and recommendations for optimization
Source: Eur J Clin Microbiol Infect Dis. 2023 Apr 24;42(7):803–18. doi: 10.1007/s10096-023-04597-7 (PMC10266996; doi:10.1007/s10096-023-04597-7)
Supplement: Supplementary file 1 — (PDF 1.44 MB) [file 10096_2023_4597_MOESM1_ESM.pdf]

Guidelines for microsatellite typing of *Toxoplasma gondii* using fifteen marker regions

Version 1.0; Date: 02.02.2023

Content

1. Preparing samples for microsatellite (MS) typing 2
1.1. DNA extraction 2
1.2. Real time PCR to specifically quantify *T. gondii* DNA concentration 2
2. MS typing workflow 2
2.1. General prerequisites and recommendations 2
2.2. Multiplex PCR 3
2.3. Loading samples to capillary sequencer 3
3. Key factors which may affect MS typing results 4
3.2. Suboptimal DNA concentration and purity 4
3.3. Varying fluorophore labeling 5
3.4. Unintended or systematic differences in primer pair synthesis 7
3.5. Effects related to capillary sequencer or size standards 7
3.6. Differences in localization of bins for automatic allele identification 7
3.7. Effects due to false interpretation of stutter peaks 7
3.8. Effects due to limited experience and due to false strain classification 8
4. General rules for reading and interpreting capillary sequencing electropherograms 8
4.2. Identification of the correct size standard peaks 9
4.3. Analyzing the electropherogram of controls 9
4.3.1. Analyzing the electropherogram of the negative control 9
4.3.2. Analyzing the electropherograms of positive controls 10
5. Recommendations for optimal reference DNAs to be used 10
6. Recommendations for lineage typing 10
7. Recommendation for reporting microsatellite results 11
8. Appearance of MS peaks and recommendation for recording results 11
9. Contact for inquiries 18
10. References 19
11. Funding 19

1. Preparing samples for microsatellite (MS) typing

1.1. DNA extraction

DNA extraction should aim to extract as much as possible *Toxoplasma gondii* specific DNA and at the same time remove inhibitory components, e.g. by using column-based DNA extraction kits or DNA extraction robots.

1.2. Real time PCR to specifically quantify *T. gondii* DNA concentration

An optimal DNA concentration (see Section 3.2.) is essential for MS typing.

- It is recommended to quantify *T. gondii* specific DNA by real time PCR.
- *T. gondii* specific real time PCR reveals a Ct value which allows the estimation of the concentration of specific *T. gondii* DNA in the sample.
- Several protocols for *T. gondii* real time PCR amplification are published (e.g. based on the 529 bp repeat or the B1 gene). Optimal protocols are those employing specific primers as well as specific probes.
- Inter-laboratory comparisons revealed that it is not possible to define optimal sample Ct values for MS typing, due to differences in real time PCR protocols and the specificities and conditions of each laboratory.

2. MS typing workflow

2.1. General prerequisites and recommendations

- Optimally, equipment, suppliers of reagents and products must be identical to those reported in the original paper (Ajzenberg et al., 2010).
- New primers have to be tested prior to their use as differences between new batches from the same or different suppliers are possible.
- The type of fluorophore has an effect on the apparent size of amplified fragments.
- To implement the method (e.g. for selecting appropriate bins for analyzing sequencing results; examples in Fig. 1-4) and as control during later typing, DNAs of *T. gondii* type I, II and III reference strains are essential (Note: Using the same type I and type II reference strains passaged in the laboratory, like RH, ME49, or PRU, may allow the identification of cross-contamination of samples by laboratory reference strain DNA. Moreover, note that in strains cultured or passaged by bioassay for a long time, the MS typing patterns may have changed [Galal et al., 2022]. Thus, ask laboratories mentioned at the end of these guidelines to provide DNAs of reference strains, for which the typing pattern is well established [see Contact section]).
- Optimally, use well established software tools to analyze your data (e.g. Gene-Mapper, Geneious Prime etc).

- If the software tool allows, use bins in your analysis in order to have repeatable results for all loci. Adjust bin location by using fragments amplified by reference DNAs.

2.2. Multiplex PCR

Reagents

- Multiplex PCR kit (e.g. 2x Qiagen Multiplex PCR Master Mix)
- 15 primer pairs (for details refer to Ajzenberg et al., 2010)
  - Forward primers labelled by 3 different fluorophores (e.g. FAM, HEX, NED as cited in Ajzenberg et al., 2010)
- Molecular grade water

Reaction mix

- Targeted final primer concentration in the reaction mix (e.g. 25 µl total volume of reaction mix): 0.2 pmol/µl each primer (Note: Primer concentration may have an effect on the analytical sensitivity of the PCR, but also on its specificity).
- Use an appropriate template volume: e.g. 1 µl for highly concentrated DNA (> 200 tachyzoite/µl and 3 or 5 µl for less concentrated samples)

Cycling conditions

- 15 min at 94 - 95°C
- 30 s at 94°C, 3 min at 61°C, and 30 s at 72°C (35 cycles)
- 30 min at 60°C

The described reagents and their concentrations are only examples, mainly based on the original published protocol (Ajzenberg et al., 2010). It is possible to use a different multiplex PCR kit and different fluorophores for primer labeling, but consider that the fluorophore can have an impact on the results. It is also possible to multiplex less than 15 targets (e.g. splitting into typing and fingerprinting markers). For specific questions, individual primer pairs can be used in singleplex PCRs.

2.3. Loading samples to capillary sequencer

Equipment and Reagents

- Formamide solution
- Size standard (labelling needs to be in accord with the fluorophores used for primers)
- Capillary sequencer (Note: Read the user guide of your capillary sequencer to confirm that all reagents for intended use are compatible with this sequencer)

PCR products can be diluted in deionized formamide, depending on the amount of template DNA. One microliter of each diluted or non-diluted PCR product should be mixed with 0.5 µl of a dye-labelled size standard (e.g. ROX 500; Applied Biosystems) and 23.5 µl of deionized

formamide. This mixture should be denatured at 95 - 100°C for 4-5 min and then electrophoresed using an automatic sequencer.

3. Key factors which may affect MS typing results

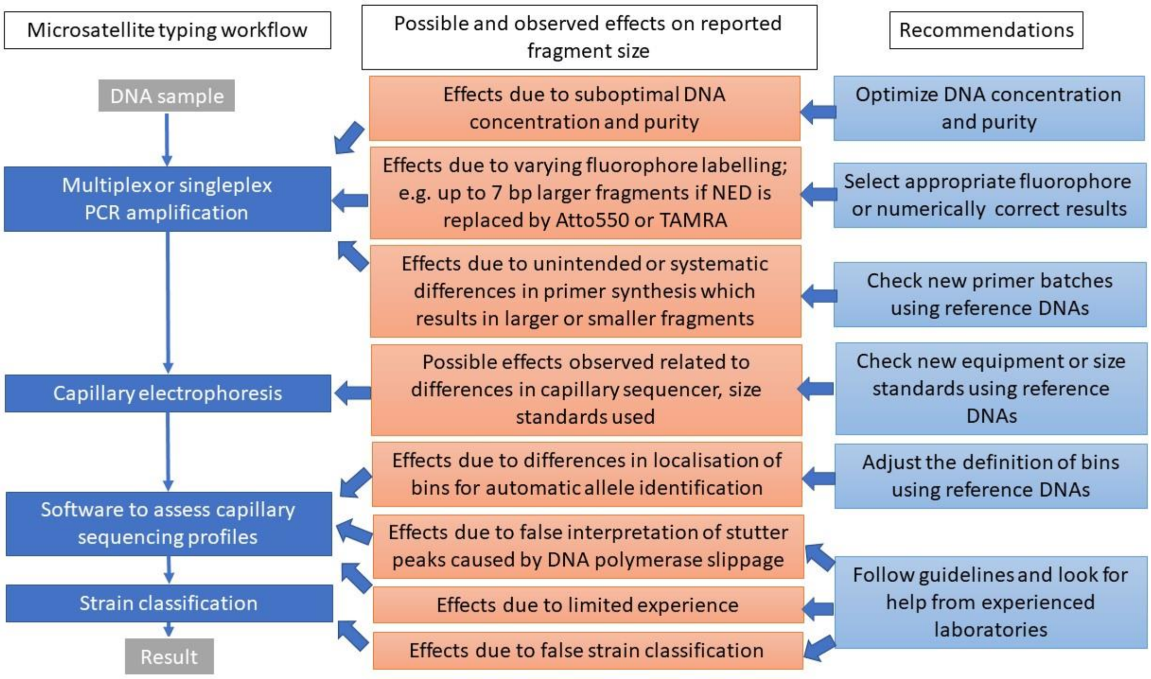

Figure 1: Putative effects on the *Toxoplasma gondii* microsatellite typing workflow responsible for laboratory-, operator-specific or unspecific differences in the microsatellite marker fragment sizes reported

3.2. Suboptimal DNA concentration and purity

An optimal DNA concentration and purity is necessary for optimal typing results. DNA concentrations can be estimated by real time PCR (Note: The type of real time PCR technique used and the specificities and conditions of each laboratory may cause differences in Ct values, even if the DNA content in the sample is identical).

Too low concentration: For samples with 0.01 ng/µl *T. gondii*-specific DNA or less, usually only part or even none of the marker regions can be amplified and used for typing.

Too high concentration: For high specific DNA concentrations, the sequencing sensor may become saturated and it is almost impossible to read the peak. For highly concentrated samples (*T. gondii* DNA concentration > 1 ng/µl), the number of non-specifically amplified fragments may increase and it might become difficult to identify the correct peaks for typing. For highly concentrated samples, so-called minus-A peaks prevail, which may complicate interpretation (**Fig. 2**).

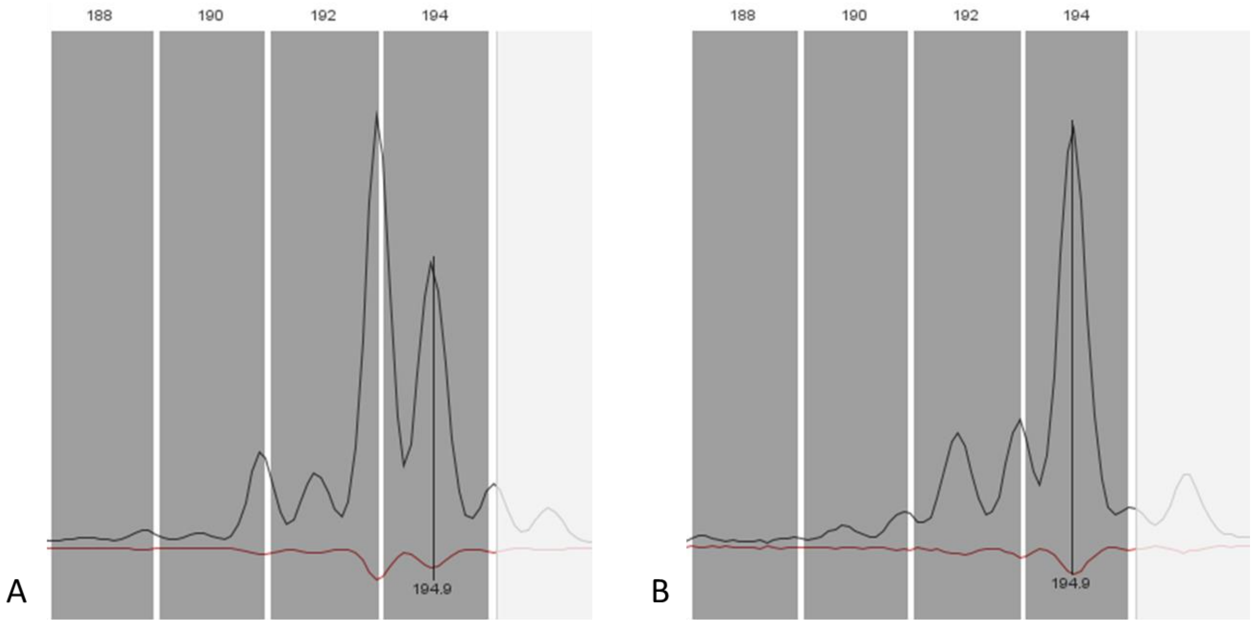

Figure 2: Illustration of the typical effect of the DNA concentration on the formation of minus-A peaks in the marker M102 of the type III reference strain NED. Bins to record fragment length results for the M102 locus are colored grey. (A) A high *T. gondii* DNA concentration caused a high minus-A peak, higher than the principal plus-A peak (194.9 bp). B: Using a 10^{-2} dilution of the original template DNA prevented the formation of a minus-A peak in this example.

3.3. Varying fluorophore labeling

Replacement of originally reported fluorophores by others may cause differences in fragment size. E.g. if NED is replaced by Atto550, up to 5 bp larger fragments are observed.

- Blue:
  - Seven markers are labelled blue including:
    - 4 typing markers: B18, M33, TUB2 and XI.1
    - 3 fingerprinting markers: N61, M48 and N83
  - Available fluorophores:
    - 6-FAM (originally reported)

- Green:
  - Five markers are labelled green including:
    - 4 typing markers: TgM-A, W35, IV.1 and B17
    - 1 fingerprinting marker: N82
  - Available fluorophores:
    - HEX (originally reported)
    - VIC: no difference to HEX-labelling reported, yet

- Yellow:
  - Three markers are labelled yellow: N60, M102 and AA
  - Available fluorophores:
    - NED (Originally reported)
    - Atto550: Differences compared to NED observed in length:
      - +4 bp for N60
      - +2 bp for M102
      - +2 bp for AA

    - TAMRA: Differences compared to NED observed in length:
      - +2 bp for N60 (example **Fig. 3**)
      - +2 bp for M102
      - +2 bp for AA

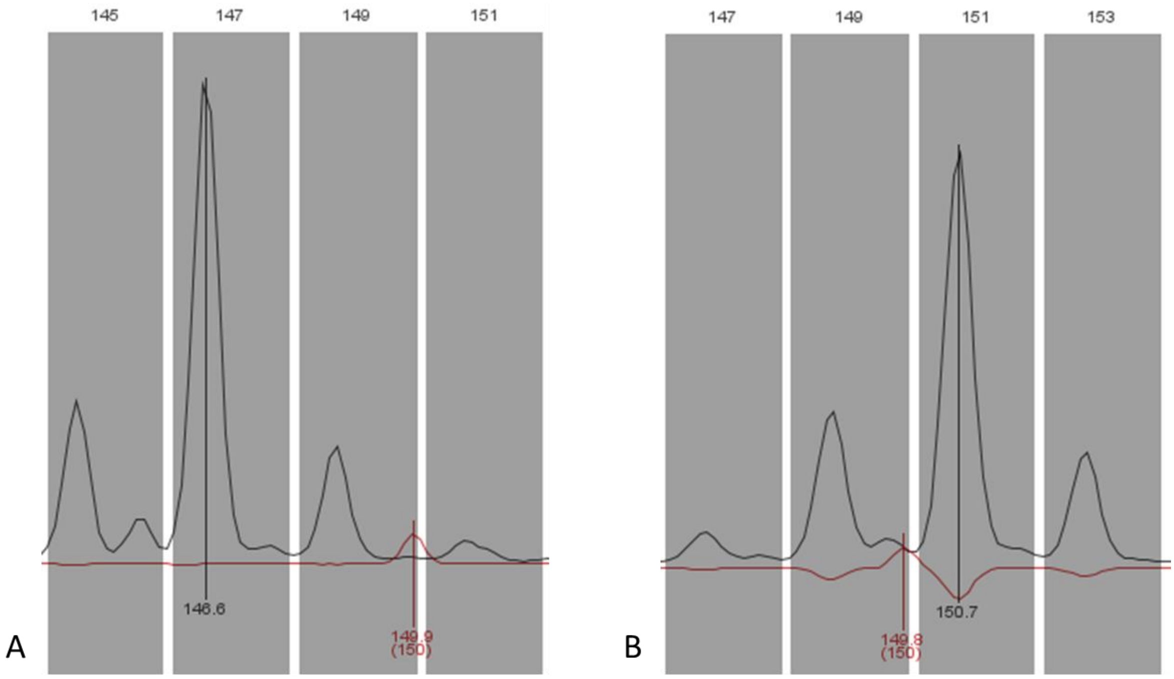

Figure 3: Example of the effect of using different fluorophores in the marker N60 for the type III reference strain NED. Bins to record fragment length results for the N60 locus are colored grey, peaks of the size standard ROX500 are colored red. If primers labelled with NED were used (A) the fragments were 4 bp smaller compared to Atto550 labelled fragments (B).

3.4. Unintended or systematic differences in primer pair synthesis

Primer pair synthesis may affect size of amplified fragments. Primer pairs with identical sequences but ordered from different suppliers resulted in different fragment sizes (**Fig. 4**). It is advised to check newly ordered primer batches using reference DNAs.

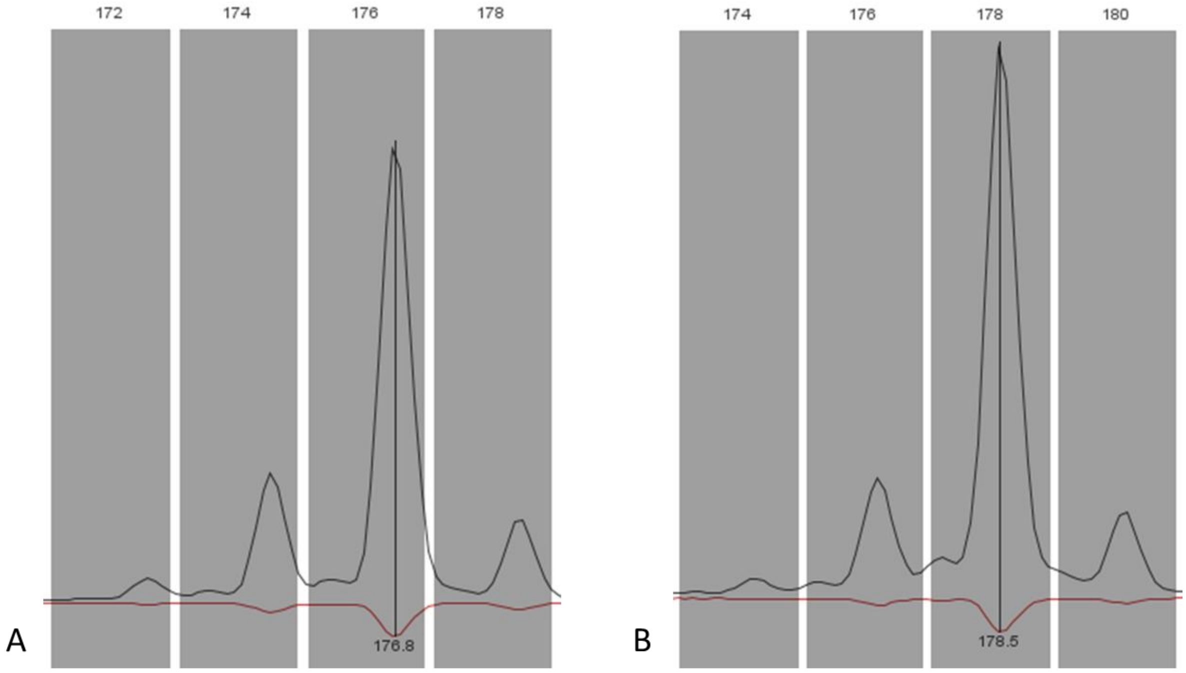

Figure 4: Example of the effect of using Atto550-labelled primers from different suppliers in the marker M102 for the type II reference strain ME49. Bins to record fragment length results for the M102 locus are colored grey. If primers with identical sequences from one supplier were used (A) the fragments were 2 bp smaller compared to fragments amplified from another supplier (B).

3.5. Effects related to capillary sequencer or size standards

Assess the effect the use of new capillary sequencing equipment or size standards may have by using reference DNAs.

3.6. Differences in localization of bins for automatic allele identification

Adjust the definition of bins using reference DNAs.

3.7. Effects due to false interpretation of stutter peaks

DNA polymerase slippage causes stutter peaks to a variable extent in the case of particular microsatellite marker regions (**Fig. 5**). Follow guidelines on the interpretation of stutter peaks (please refer to Section 7) and look for help from experienced laboratories.

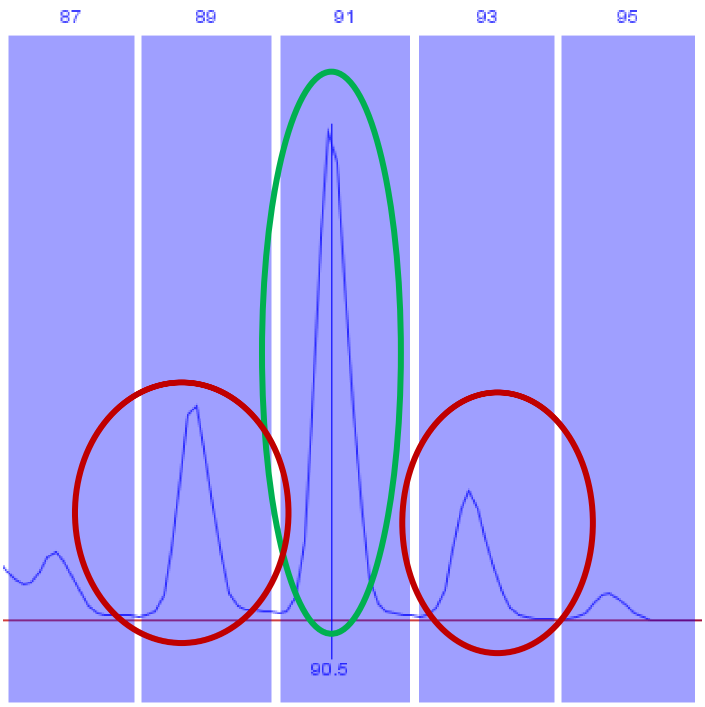

Figure 5: Example of non-specific stutter peaks (Red), with the true peak for this marker N61 (Green). Bins to record fragment length results for the N61 locus are colored light blue.

3.8. Effects due to limited experience and due to false strain classification

Limited experience in interpretation of capillary sequencer profiles may mean that unspecific peaks are recorded while specific peaks are missed. This may cause false strain classification. Follow guidelines and look for help from experienced laboratories.

4. General rules for reading and interpreting capillary sequencing electropherograms

Each MS typing experiment should include positive controls consisting of reference DNAs. Please refer to optimally characterized reference DNAs mentioned in this guideline (Type I, Type II, Type III, Africa1). A negative control needs to be included too (e.g. water or any appropriate DNA related to the sample [i.e. DNA from the same host species or the same sample matrix without Toxoplasma DNA]).

For reading the profiles please always follow the same order:

- First, check out the size standard peaks for each of the genotyped samples, beginning with the positive and negative controls. Please follow the user guide of your capillary sequencer.
- In a second step, analyze the profiles of negative and positive controls.

- In a third step, analyze profiles of test samples. Visualize and assess the electropherograms of test samples individually, fluorophore by fluorophore and always compare peak characteristics observed in individual samples to those of the negative and positive controls (see below).

4.2. Identification of the correct size standard peaks

For the correct estimation of fragment sizes of amplified microsatellite markers, it is essential to identify all size marker peaks correctly (**Fig. 6**). Size estimates provided for these peaks need to be compared to those provided from the supplier of the size standard. Capillary sequencer user guides may recommend excluding particular markers from fragment length analysis. The intensity of the size standard peaks should be 30% to 100% of the intensity of the sample peaks.

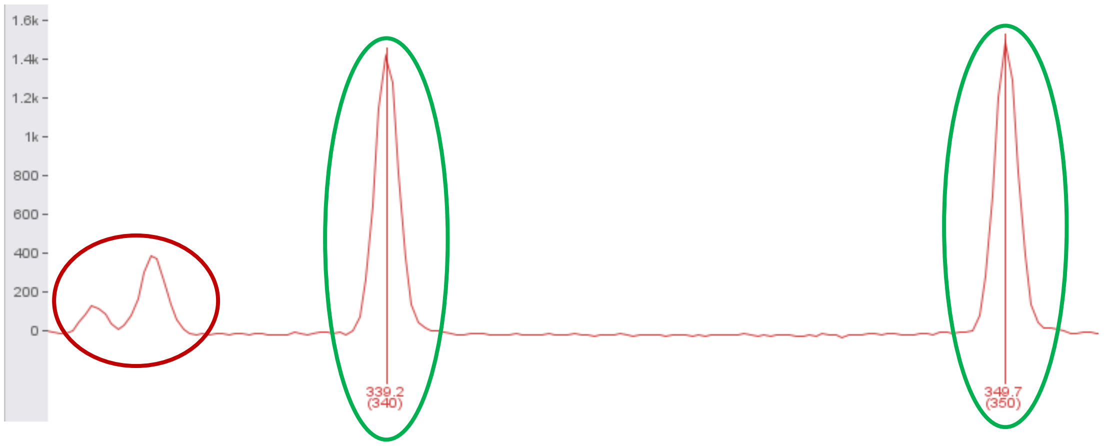

Figure 6: Correct (Green) and incorrect (Red) aspects of the size standard (ROX500) peaks.

4.3. Analyzing the electropherogram of controls

Peaks of controls and also those of the unknown samples should have about the same height or up to three times the height of size standard peaks. If they are too high, there is too much *T. gondii* DNA. This may result in the emergence of the so-called Minus A-Peaks. For optimal results, repeat the experiment with diluted template DNA or dilute the PCR products. To avoid experiment repetitions, adjust the amount of specific DNA beforehand based on real time PCR results.

4.3.1. Analyzing the electropherogram of the negative control

Look at the profile of the negative control:

- Confirm that MS peaks are absent. If there are MS peaks this could be an indication of carry-over or cross contamination.
- Look out for non-specific peaks (e.g. so-called “injection peaks”). This may help to identify non-specific peaks in the profiles of the unknown samples.

4.3.2. Analyzing the electropherograms of positive controls

Look at the profile of the positive control(s):

- Confirm that the peaks identified match the values expected for reference DNA.
- Confirm location of bins.
- Look for non-specific peaks, as described for the negative control.
- If there are differences in the results, to those expected, consider potential reasons as mentioned in Section 3.

5. Recommendations for optimal reference DNAs to be used

For reliable MS typing, and especially when you start using the MS genotyping technique for the first time, choose some of the DNAs from the following isolates to include them in each run of MS typing: GT1 or RH (Type I), ME49 or PRU (Type II), CTG, VEG or NED (Type III). Later, one *T. gondii* Type II and one Type III reference DNA appears to be sufficient as controls for individual runs. As mentioned earlier, please note that, during long-time cultivation or passaging of strains, minor changes in the MS patterns may occur. Thus, it is recommended to ask the authors of these guidelines for well characterized reference DNAs from their stocks.

6. Recommendations for lineage typing

For lineage typing please refer to **Table 1**.

Table 1: Expected values for lineage typing markers for different reference strains (Blue, labelled with 6-FAM; Green, labelled with HEX) (Galal et al., 2022)

MS genotype	B18	M33	TUB2	XI.1	TgM-A	W35	IV.1	B17
Type I	160	169	291	358	209	248	274	342
Type II	158	169	289	356	207	242-4	274	336
Type III	160	165	289	356	205	242	278	336
Africa 1	160	165	291	354	205	248	274	342
Africa 3	160	165	291	354	207	242	278	342
Africa 4	156	165	291-3	354	203	242	274	336
Caribbean 1	162	165	291	356	205	242	278	342
Caribbean 2	162	165	291	356	205	242	278	336
Caribbean 3	162	165	289	356	205	242	278	336
Chinese 1	160	169	293	354	211	242	274	336
Type 12*	158-60-62	169	289	356-62	207-9-11	242	274	336

* Type 12 has a complex classification, probably because of more than a single population within this lineage

In the case of minor deviations (up to 2 bp) from the expected values in a single MS marker, it is advised to designate the observed genotype as Type-variant. Such variants may often not represent recombinants or unclassified strains but variants of known lineages. To confirm recombination or the observation of an unclassified strain, it is necessary to use other techniques, optimally whole genome sequencing. Multi locus sequence typing (MLST) may also help.

7. Recommendation for reporting microsatellite results

Reporting microsatellite results, e.g. in publications, requires that essential details of genotyping MS markers are mentioned:

Methods

- A statement on primer pair sequences (e.g. a reference)
- The fluorophores used for individual primer pairs
- The supplier of the primers
- The size marker applied and which of the markers were used to assess fragment size
- Treatment of amplified fragments prior to loading capillary sequencer
- Capillary sequencing equipment
- Software used to asses sequencing profiles and a brief description on details (loci, bins)
- *Toxoplasma gondii* reference DNAs used in individual runs, including their origin/provider

Results

- MS typing results and Ct values for all samples
- MS typing results for reference DNAs

8. Appearance of MS peaks and recommendation for recording results

In the following table, the appearance of peaks in *T. gondii* MS typing and a number of recommendations on how to record MS fragment sizes based on capillary sequencing are listed (**Table 2**). Please note, that these peak profiles displayed are only examples. The appearance of peaks is often similar for a given marker, but may change from one experiment to another. Among others, potential reasons are differences in template DNA concentration, the capillary sequencing device and the type of PCR applied, i.e., multiplex or simplex. Note, that stuttering of DNA polymerase may result in multiple fragment sizes and in a “Hedgehog”-like appearance of the sequencing profile.

Table 2: Exemplary appearance of peaks in *T. gondii* MS typing and recommendations on how to record MS fragment sizes based on capillary sequencing results. The appearance of the profiles can vary, e. g. due to different concentrations of the samples. In these examples, the correct peaks are marked with an arrow.

**Marker (lineage typing markers are bold)**	**Recommend-ations for recording**	**Example (ME49) for peak appearance (Capillary sequencer: Hitachi 3500 Genetic Analyzer, Analyzing software: Geneious Prime)**	**Example (ME49) for peak appearance (Capillary sequencer: 3130xl Genetic Analyzer, Analyzing software: Gene-Mapper) Acronyms: al = recorded size, sz = original size, ht = height of the peak**
N61	- Odd value<br>- This marker is prone to stuttering (“Hedgehog” look); consider the last high peak before the peak height starts to decrease	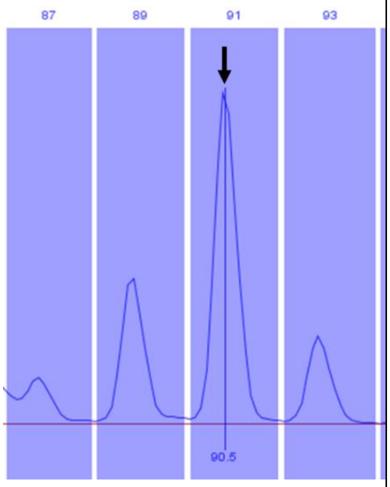	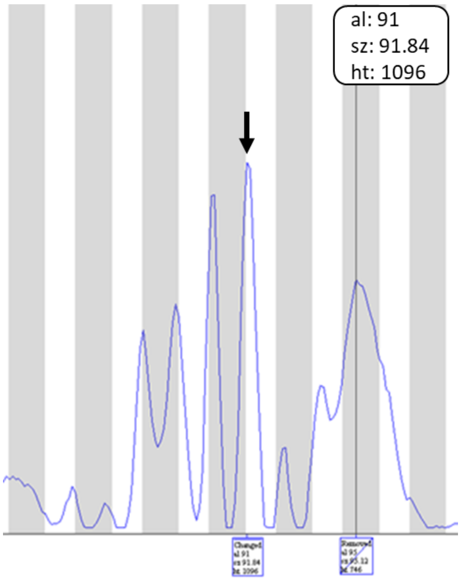
**B18**	- Always record an even value	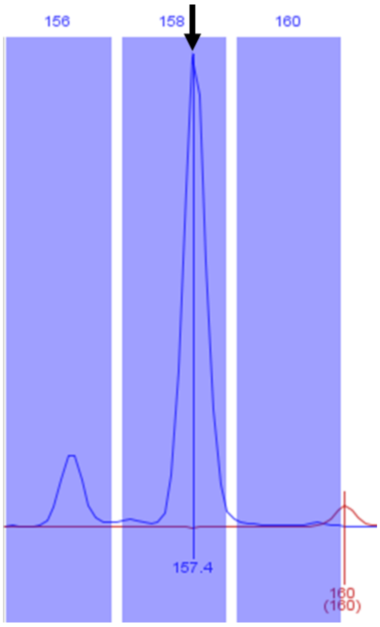	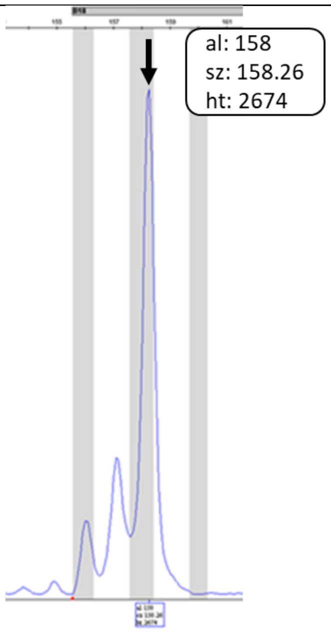

**M33**	- Always record an odd value<br>- In case of a double peak, located in two different bins, choose the 2nd one	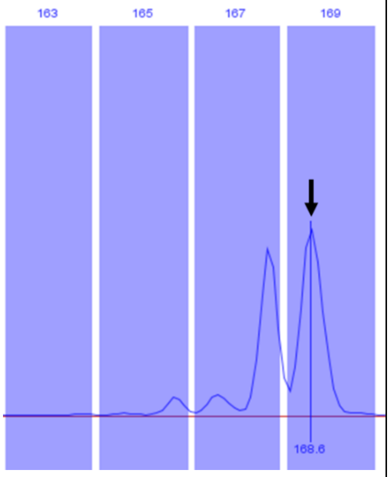	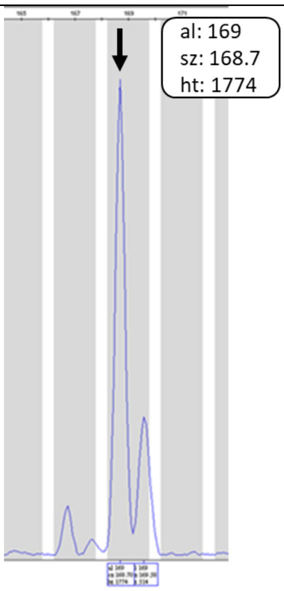
M48	- Always record an odd value<br>- In the case of a double peak, located in two different bins, choose the 2nd one	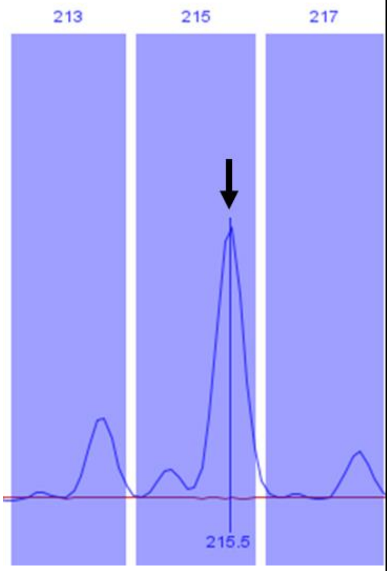	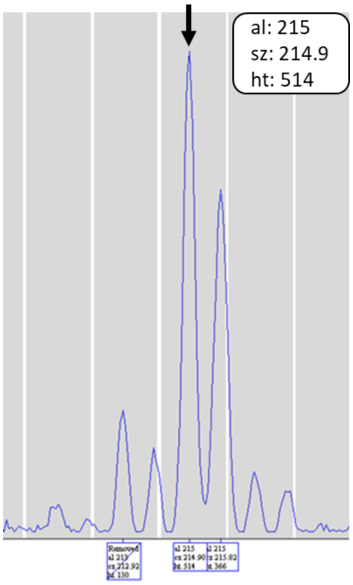
**TUB2**	- Always record an odd value<br>- In the case of a double peak, located in two different bins, choose the 2nd one	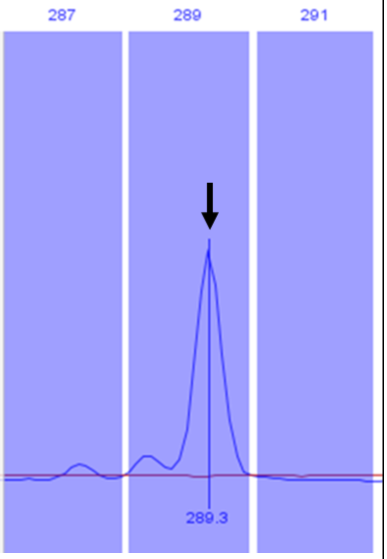	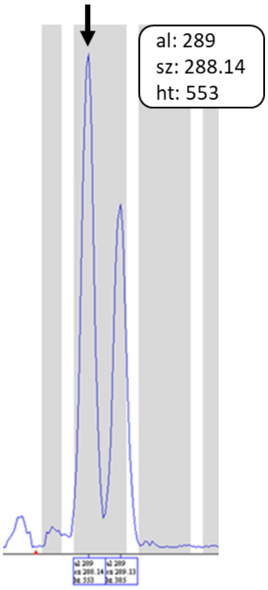

N83	- Always record an even value	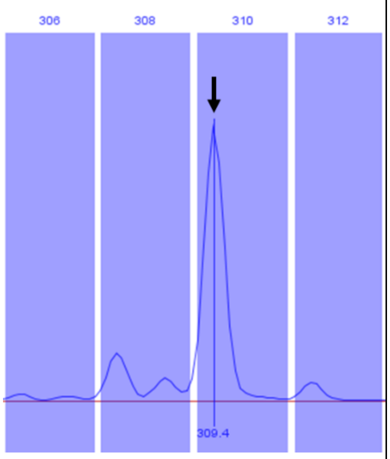	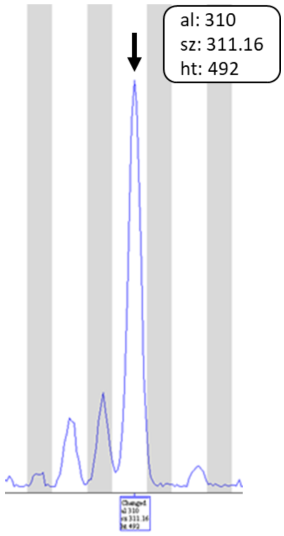
**XI.1**	- Always record an even value<br>- In the case of a double peak, located in two different bins, choose the 2nd one (it may be weak or absent in type II)	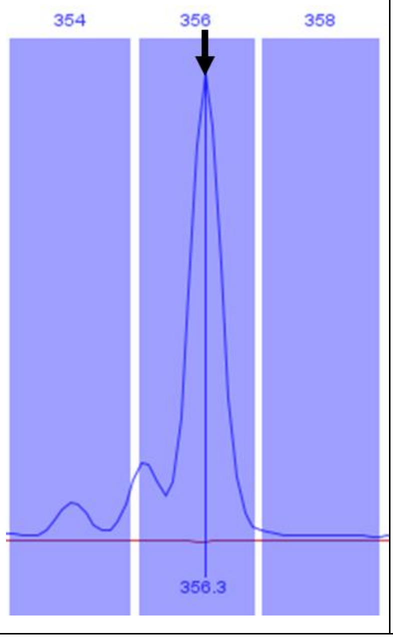	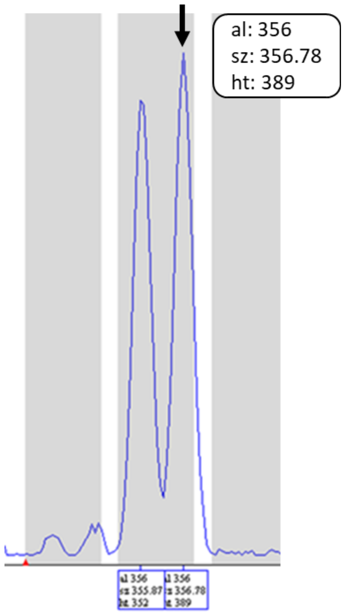

N82	- Always record an odd value<br>- Usually, there is only a single peak	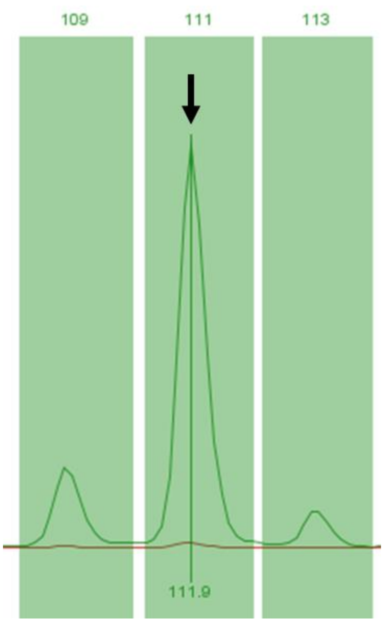	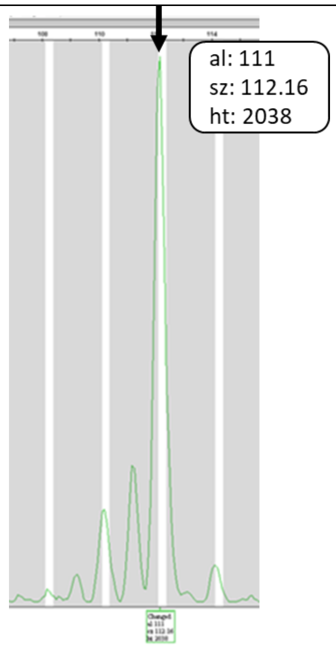
**TgM-A**	- Always record an odd value<br>- In the case of a double peak, located in two different bins, choose the 2nd one	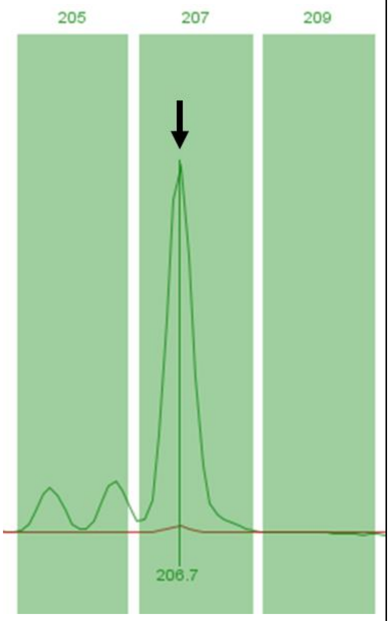	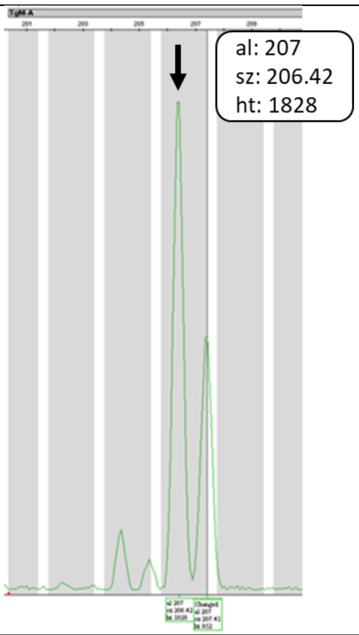
**W35**	- Always record an even value<br>- In the case of a double peak, located in two different bins, choose the 2nd one	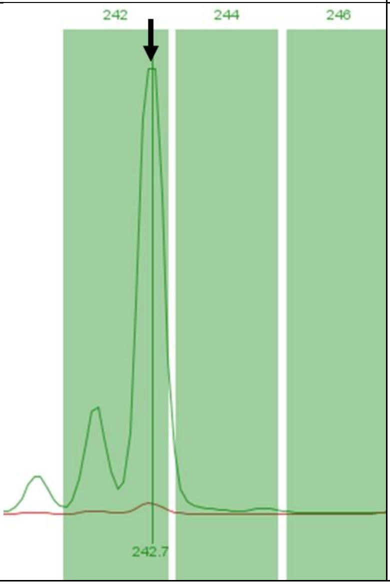	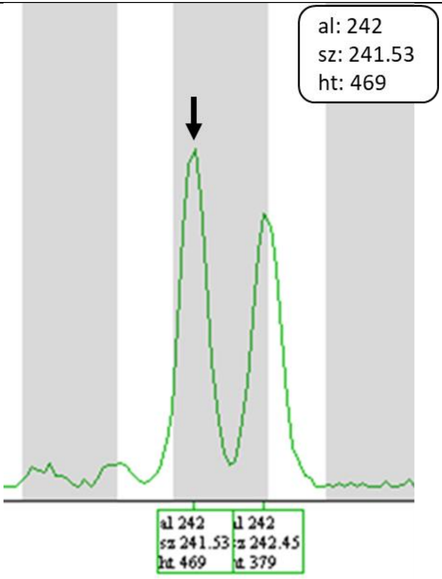

**IV.1**	- Always record an even value<br>- In the case of a double peak, located in two different bins, choose the 2nd one	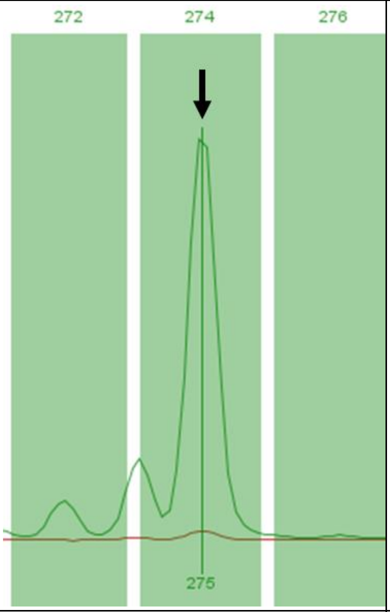	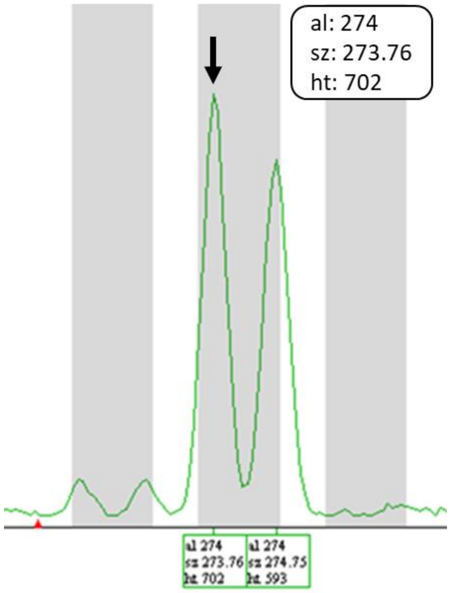
**B17**	- Always record an even value<br>- In the case of a double peak, located in two different bins, choose the 2nd one	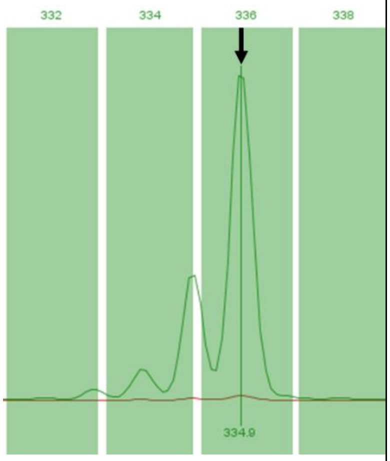	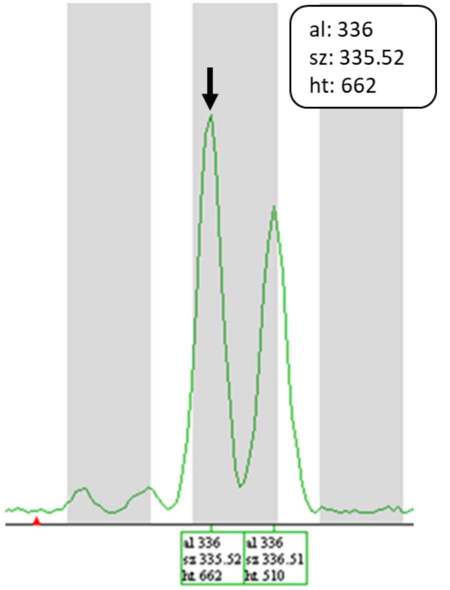

N60	Dye: NED<br>- Always record an even value when < 145 and an odd value when ≥ 145<br><br>Dye: Atto550<br>- Always record an even value when < 149 and an odd value when ≥ 149<br><br>Dye: TAMRA<br>- Always record an even value when < 147 and an odd value when ≥ 147	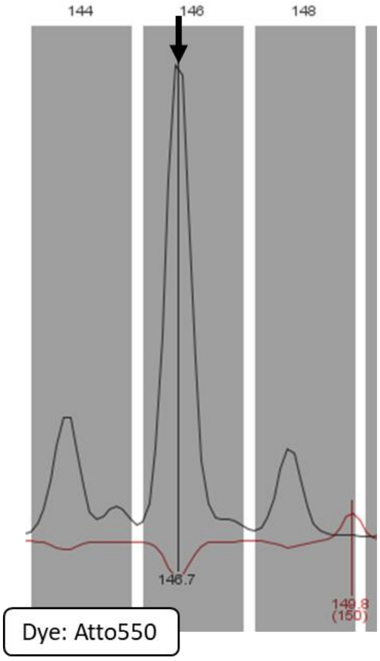	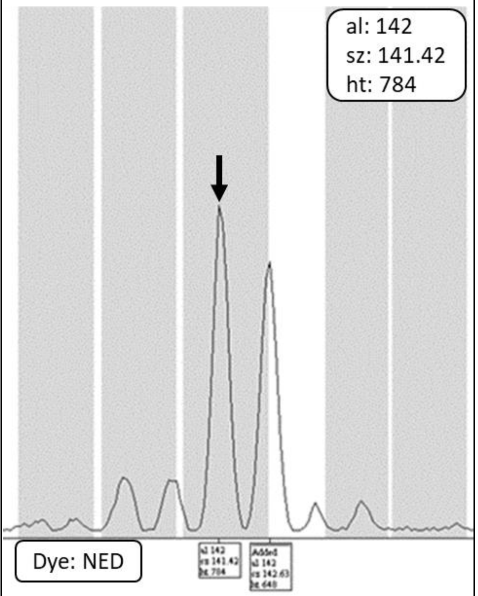
M102	- Always record an even value<br>- In the case of a double peak, located in two different bins, choose the 2nd one	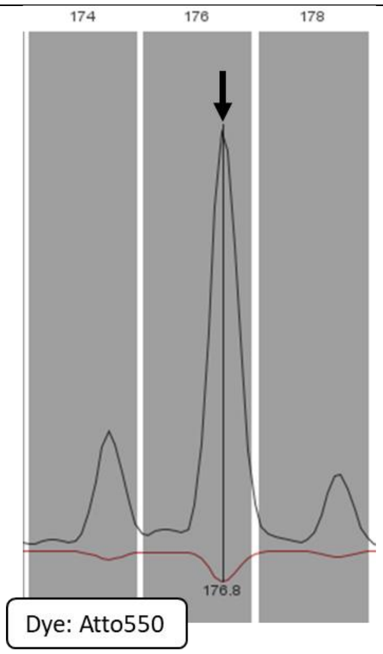	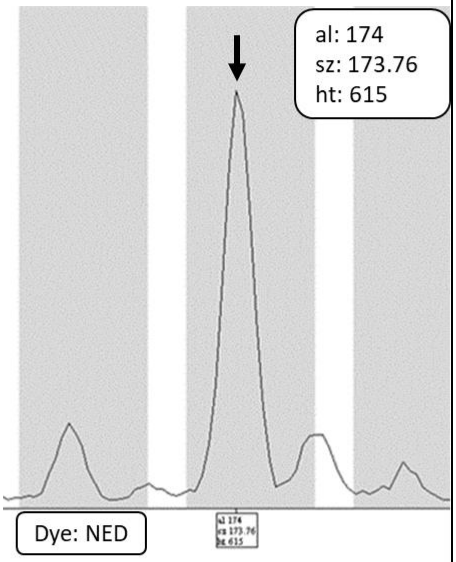

AA	- Always record an odd value<br>- This marker is susceptible to stuttering.<br>- Select the highest peak when stuttering is observed (“Hedgehog” look)	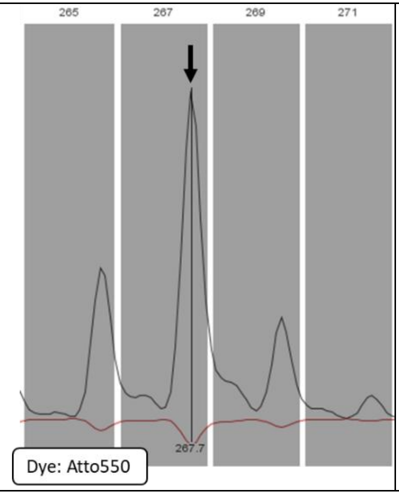	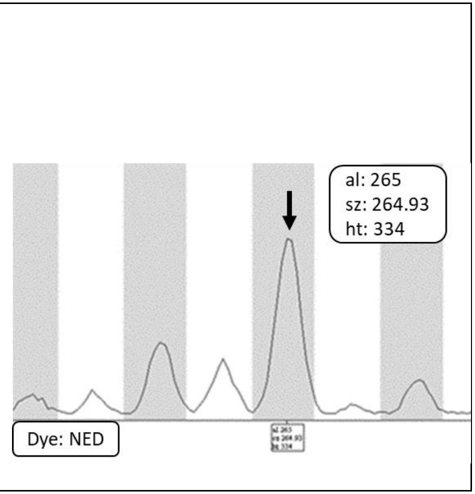

9. Contact for inquiries

Aurélien Mercier, aurelien.mercier@unilim.fr
Inserm U1094, IRD U270, EpiMaCT - Epidemiology of chronic diseases in tropical zone
Faculté de Médecine
2, rue du Dr Marcland, 87025 Limoges, France
Tél : +33 5 19 56 42 65
http://www.unilim.fr/ient

Gereon Schares, gereon.schares@fli.de
Friedrich-Loeffler-Institut
Federal Research Institute for Animal Health
Südufer 10, 17493 Greifswald - Insel Riems, Germany
Tel: +49 38351 71544/1694/1695/1658/1677 | Fax: +49 38351 7 1526, 1174
https://www.fli.de

Rafael Calero-Bernal, r.calero@ucm.es
Complutense University of Madrid
Ciudad Universitaria s/n, Madrid 28040, Spain
Tel: +34913944095
https://ucm.es/saluvet

Clare Hamilton, clare.hamilton@moredun.ac.uk
Moredun Research Institute, Pentlands Science Park
Bush Loan, Penicuik, Midlothian EH26 0PZ, Scotland
Tel: +44 (0)131 445 5111 | Fax: +44 (0)131 445 6111
https://www.moredun.org.uk

Pikka Jokelainen, pijo@ssi.dk
Infectious Disease Preparedness
Statens Serum Institut
Artillerivej 5, 2300 Copenhagen S, Denmark
Tel: +45 32688679
https://en.ssi.dk

10. References

(1) Ajzenberg D., Collinet F., Mercier A., Vignoles P., et Dardé M.-L. Genotyping of *Toxoplasma gondii* isolates with 15 microsatellite markers in a single multiplex PCR assay. Journal of Clinical Microbiology. 2010 Dec, 48 (12): 4641-45. doi:10.1128/JCM.01152-10.

(2) Galal L, Ariey F, Gouilh MA, Dardé ML, Hamidović A, Letourneur F, Prugnolle F, Mercier A. A unique *Toxoplasma gondii* haplotype accompanied the global expansion of cats. Nat Commun. 2022 Oct 1;13(1):5778. doi: 10.1038/s41467-022-33556-7.

11. Funding

This work was supported by funding from the European Union's Horizon 2020 Research and Innovation programme under grant agreement No 773830: One Health European Joint Programme, TOXOSOURCES project, by funds from the French Agence Nationale de la Recherche (ANR project IntroTox 17-CE35-0004) and funds from the Scottish Government Rural and Environment Science and Analytical Services Division (RESAS).
